# Efficacy of long-term oral nutritional supplementation with dietary counseling on growth, body composition and bone mineralization in children with or at risk for undernutrition: a randomized controlled trial

**DOI:** 10.1186/s12937-025-01133-5

**Published:** 2025-07-14

**Authors:** Mandy Y. L. Ow, Nga Thuy Tran, Yatin Berde, Tu Song Nguyen, Van Khanh Tran, Morgan J. Jablonka, Geraldine E. Baggs, Dieu T. T. Huynh

**Affiliations:** 1 Abbott Nutrition R&D Asia-Pacific Center, Abbott Laboratories, Abbott Manufacturing Singapore Pte Ltd, 20 Biopolis Way, #09-01/02 Centros Building, Singapore, 138668 Singapore; 2 Department of Micronutrients, National Institute of Nutrition, Hanoi, Vietnam; 3 Mars Group Inc., Mumbai, India; 4 Department of General Planning, National Institute of Nutrition, Hanoi, Vietnam; 5 Abbott Nutrition R&D, Abbott Laboratories, Columbus, OH USA

**Keywords:** Catch-up growth, Stunting, Underweight, Wasting, Lean mass, Fat mass

## Abstract

**Background:**

Impaired growth, accompanied by low lean mass and poor bone mineralization in undernourished children, is linked to adverse short- and long-term health outcomes. Oral nutritional supplements (ONS) promote catch-up growth, but their efficacy in improving lean mass and bone mineralization remains uninvestigated. This study aims to compare the efficacy of long-term ONS with dietary counseling (DC) versus DC alone on growth, body composition, bone mineralization, and health outcomes in children with or at risk of undernutrition.

**Methods:**

Children (*n* = 330) aged 24–60 months with WHO Growth Standard z-scores of weight-for-age < − 1, height-for-age < − 1, and weight-for-height < 0 were randomized in a multisite controlled trial to receive two servings of a complete and balanced ONS formula with DC, or DC-only, for 240 days. Anthropometric measurements, dietary intake, and parent-reported measures of illness-related and other health outcomes were assessed at baseline and days 30, 120, and 240. Dual X-ray absorptiometry–assessed body composition and bone mineralization, and nutritional blood biomarkers were measured at baseline and day 240.

**Results:**

ONS supplementation augmented growth in height and weight through day 240, with increasing between-group differences over visits (*P* < 0.01 for treatment-by-visit interaction in height, weight, height-for-age and weight-for-age z-scores). Energy and protein intake levels were 26% and 22% higher, respectively, in the ONS + DC compared to the DC-group at day 240 (both *P* < 0.001). The ONS + DC group also had a higher lean mass index of 11.06 (0.05) versus 10.92 (0.05) kg/m2 (*P* = 0.048) and total body less head bone mineral density of 0.407 (0.003) versus 0.399 (0.003) g/cm2 (*P* = 0.03) at day 240, with no differences in fat mass index compared to DC. The ONS + DC group also had better serum vitamin D and K status, fewer sick and missed school days, better parent-reported sleep habits, appetite, energy, and physical activity levels versus DC-group (all *P* < 0.05).

**Conclusion:**

Adding ONS to DC for 8 months improved linear catch-up growth and supported quality growth, as evidenced by greater lean mass and bone mineral accretion. These findings, alongside parent-reported improvements in child health, suggest that improved nutrient intake with ONS improves multiple domains of child health and well-being.

**Trial registration:**

This clinical trial was registered on ClinicalTrials.gov (registration number: NCT05239208) on 14 February 2022.

Video Abstract

**Supplementary Information:**

The online version contains supplementary material available at 10.1186/s12937-025-01133-5.

## Background

Childhood undernutrition is associated with physical, cognitive, and metabolic developmental impairments that may have long-term health consequences [1, 2]. Beyond impaired growth, undernourished children often exhibit altered body composition (BC) [3] and reduced bone mineralization (BM) [4–7]. Lean and fat mass serve distinct biological functions and influence outcomes differently in these children [3, 8]. Low muscle or lean mass is associated with higher mortality and poorer clinical outcomes in the short term [9], and may elevate noncommunicable disease risks in the long term [10]. While fat mass is essential for immune function, energy, and metabolic homeostasis [3, 11], excess weight and fat gain during recovery can lead to adverse long term effects, including obesity and increased noncommunicable disease risks [3, 12]. Additionally, low bone mineral density (BMD) is associated with a higher fracture risk in childhood [13]. If uncorrected, this likely leads to a lower peak bone mass in adulthood [14, 15] which is associated with higher lifetime risks of osteoporosis and fracture [16].

Providing adequate and the right balance of nutrients is crucial to restoring physiological normality beyond weight gain when addressing undernutrition in children [17]. Lean tissue synthesis requires a relatively higher nutrient density than adipose tissue accretion, and skeletal tissue may need specific nutrients concentrated in cartilage and bone (eg, sulfur-rich amino acids, vitamins D and K, calcium, etc.) [17]. An imbalanced nutrient intake, particularly a deficiency in growth nutrients, can result in excess energy being deposited as adipose tissue rather than being utilized for lean tissue synthesis [3, 17, 18]. Therefore, evaluating BC and BM provides valuable insight on the quality of growth in terms of types of tissues accreted during nutritional supplementation. Oral nutritional supplements (ONS) are designed to promote catch-up growth in undernourished children [19–25]. However, there is a paucity of studies examining the impact of ONS or supplementary food on growth quality, specifically BC and BM, in addition to traditional anthropometric metrics such as height and weight in this population.

Supporting Pediatric Growth and Health Outcomes (SPROUT) is a 240-day randomized controlled trial (RCT) comparing the efficacy of a complete and balanced ONS with dietary counseling (DC) versus DC alone in children aged 24 to 60 months with or at risk of undernutrition. The study primary endpoint, change in weight-for-age z-score (WAZ) at day 120, was achieved and previously reported with other 120-day outcomes [26]. Here, 240-day outcomes for growth, BC and BM assessed by dual-energy X-ray absorptiometry (DXA), nutritional blood biomarkers, and other child health measures are reported.

## Methods

### Study design

SPROUT is a community-based, randomized, open-label, parallel-group, controlled interventional trial spanning 240 days, involving one primary center and seven preschool satellite sites in Vietnam. The study was conducted according to the guidelines of the Declaration of Helsinki and approved by the independent ethics committee/institutional review board of the National Institute of Nutrition in Hanoi, Vietnam (approval code: 1251/VDD-QLKH). The study was conducted and reported according to the Consolidated Standards of Reporting Trials (CONSORT) guidelines (CONSORT checklist in Supplementary File S1) and was prospectively registered at ClinicalTrials.gov (NCT05239208). Protocol details and 120-day study outcomes have been published [26]. Briefly, between January and April 2022, 330 children aged 24–60-months old, with or at risk for undernutrition (defined as WAZ < − 1, height-for-age z-score (HAZ) < − 1, and weight-for-height-z score (WHZ) < 0 according to the 2006 WHO Growth Standards) were enrolled [27]. They were excluded if born pre-term (birth before 37 weeks of gestation, as reported by a parent), had a birth weight < 2500 g or > 4000 g, had current acute or chronic infections, diagnosed with a clinically significant medical condition, or clinically significant nutritional deficiency requiring specific treatment with another nutritional supplement (other than the study product) in the opinion of the investigator. All parents provided written informed consent for participation.

### Study procedures

Eligible participants were stratified by sex and age (24 to ≤ 36 months and > 36 to ≤ 60 months) and randomized 1:1 to the intervention group (ONS + DC) or control group (DC only). Randomization schedules were computer generated using a biased-coin minimization approach. As an open-label study, neither participants nor all investigators and study team were blinded to treatment allocation. Nevertheless, measures were taken to reduce bias, including provision of study product in plain foil packaging to avoid disclosure of ONS identity and nutritional composition, and blinding of clinicians/nurses who performed study outcome assessments wherever possible to reduce assessor bias [28]. All study procedures except the DXA scans were completed at the satellite sites (preschools). DXA scans of the whole body, lumbar spine, and bilateral total hip were performed at a clinic facility. Outcomes were assessed at baseline and days 30, 120, and 240, except for DXA scans and blood sampling (baseline and day 240) and lower leg length (baseline and days 120 and 240). DC was provided to all parents at baseline, and days 30, and 120. Dietary intake was assessed using the 24-h dietary recall method, and DC incorporated techniques to improve diet quality and to help the child meet nutrient requirements. DC content was based on local food-based dietary guidelines and the local food pyramid [29, 30]. With respect to milk and dairy products, the DC-only group was encouraged to continue with their current milk intake, and the ONS + DC group was counseled to consume ONS in replacement of their current milk intake, added onto but not replacing other components of their usual dietary intake. Two servings of ONS were administered by teachers at preschool on weekdays and by parents at home on weekends or holidays for 240 days. One serving of ONS (PediaSure; Abbott Laboratories, Vietnam) provided 226 kcal of energy, 6.74 g of protein, 8.81 g of fat, and 29.47 g of carbohydrates (Supplementary Table S1). Use of any non-study ONS (defined as formulas with an energy density ≥ 1 kcal/mL, containing protein, carbohydrate, and/or fat, as well as a wide range of micronutrients to supplement or use as the sole source of nutrition), as well as any product from Abbott Nutrition other than the study product, was not allowed in either study group.

### Outcomes

The primary study endpoint was change in WAZ at day 120. Secondary outcomes measured at days 30, 120, and 240 included anthropometry (weight, height, WAZ and weight-for-age percentile (WAP), HAZ and height-for-age percentile (HAP), WHZ and weight-for-height percentile (WHP), body mass index-for-age z-score and percentiles, and mid-upper-arm circumference for-age z-score and percentiles based on the WHO Growth Standards) [27], as well as dietary intake assessed using the 24-h dietary recall method. Energy and macronutrient intake and nutrient adequacy were assessed. Nutrient adequacy was defined as meeting 77% of the daily recommended nutrient intake (RNI) [31]. Age- and sex-specific RNIs from Vietnamese Recommended Dietary Allowances were used. [32] Other outcomes included height-for-age and weight-for-age differences, arm anthropometric indices, lower leg length, DXA-assessed BC and BM, handgrip strength, nutritional blood biomarkers (capillary blood hemoglobin, serum total 25-hydroxyvitamin D, serum albumin, serum undercarboxylated osteocalcin (ucOC), serum carboxylated osteocalcin (cOC), and serum amino acids), parent-reported illness-related outcomes, attentional focus, sleep habits, physical activity, energy, appetite, and parental satisfaction with child’s health. Details of exploratory endpoints and their assessment methods and adverse event (AE) collection are listed in Supplementary Table S2.

### Statistical analysis

Sample size was calculated based on the primary endpoint and described previously [26]. The intent-to-treat (ITT) population included all randomized participants in the control group and all randomized participants who received at least one study feeding in the intervention group. Continuous endpoints measured at ≥ 3 timepoints were analyzed with repeated-measures analysis of covariance (ANCOVA) with main effects for site, treatment, sex, and visit; interaction effects of treatment-by-sex and treatment-by-visit; and age and baseline values of the dependent variables as covariates. Continuous endpoints, as well as change from baseline at each timepoint, were analyzed with ANCOVA with the same factors and covariates (excluding visit and treatment-by-visit terms). BC and BM endpoints assessed with DXA included additional covariates of height and weight due to the known influence of body size on DXA parameters [33], and bone mineral apparent density (BMAD) was computed for lumbar spine scans [34]. Categorical outcomes were analyzed using chi-squared tests; logistic regression at each timepoint with similar factors as in ANCOVA models; or repeated measures generalized estimating equations with the same factors and covariates plus visit and treatment-by-visit terms. For the illness frequency outcome, univariate analysis was first performed on covariates known to affect child immunity (covariates are listed in Supplementary Table S2). Covariates were added and retained in the final model if *P* < 0.10. Based on these criteria, duration of breastfeeding was identified and added to the final models of all illness-related outcomes. All tests of hypotheses were two-sided 0.05 level tests, except tests for interaction, which were two-sided 0.10 level tests.

## Results

### Participant characteristics

**Fig. 1:**
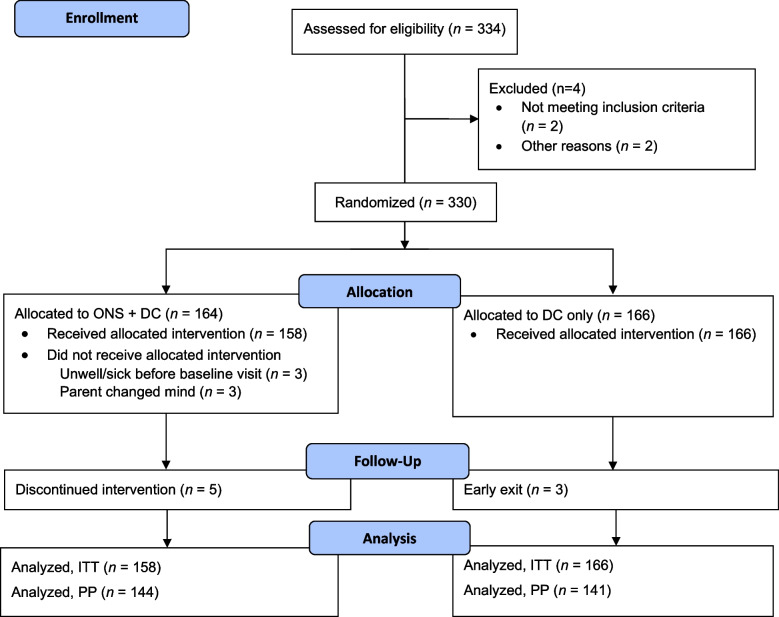
CONSORT flow diagram for wave 2 of the SPROUT study. AE, adverse event; CONSORT, Consolidated Standards of Reporting Trials; DC, dietary counseling; ITT, intention-to-treat; ONS, oral nutritional supplement; PP, per-protocol; SPROUT, Supporting Pediatric Growth and Health Outcomes

A total of 330 children were randomized to ONS + DC (*n* = 164) or DC only (*n* = 166); 6 participants in the ONS group did not receive any ONS and were excluded from the ITT population (Fig. 1). All results presented are for ITT population (*n* = 324). Overall study discontinuation rate was very low (2.5%) with 3.2% and 1.8% in the intervention and control groups, respectively. Key baseline characteristics are listed in Table 1; detailed characteristics have been published previously [26]. Briefly, mean participant age was 46.5 months at enrollment and 48.5% were male. Approximately two-thirds of the study population were mildly stunted or mildly underweight at baseline, with most of the remaining children being moderately stunted or moderately underweight. Very few children (< 5%) were severely stunted, underweight, or wasted. Additionally, 49.7% of the study population had mild wasting, whereas 41.9% did not have wasting (WHZ ≥ − 1 and < 0) (Table 1).

**Table 1:** Key baseline sociodemographic and anthropometric characteristics of participants

Child characteristics	ONS + DC (*n* = 158)	DC only (*n* = 166)
Age (months), mean (SE)	46.8 (0.7)	46.3 (0.6)
Age categories, n (%)		
> 24 to ≤ 36 months	19 (11.4)	18 (11.4)
> 36 to ≤ 60 months	140 (88.6)	147 (88.6)
Sex, n (%)		
Male	76 (48.1)	81 (48.8)
Female	82 (51.9)	85 (51.2)
Anthropometry, LSM (SE)^a^		
Weight-for-age (z-score)	− 1.91 (0.05)	− 1.85 (0.04)
Height-for-age (z-score)	− 1.84 (0.04)	− 1.86 (0.04)
Weight-for-height (z-score)	− 1.23 (0.05)	− 1.12 (0.05)
BMI-for-age (z-score)	− 1.07 (0.05)	− 0.96 (0.05)
MUAC-for-age (z-score)	− 1.23 (0.05)	− 1.19 (0.05)
Height-for-age difference (cm)	− 7.65 (0.18)	− 7.76 (0.18)
Gestational age (weeks)	38.5 (0.1)	38.5 (0.1)
Exclusive breastfeeding (months)	5.14 (0.13)	5.33 (0.13)
Total breastfeeding^*b*^ (months)	8.82 (0.37)	8.74 (0.37)
Nutritional status		
Stunting		
Normal	1 (0.6)	0 (0.0)
Mild	107 (67.7)	106 (64.6)
Moderate	45 (28.5)	56 (34.1)
Severe	5 (3.2)	2 (1.2)
Underweight		
Normal	0 (0.0)	1 (0.6)
Mild	111 (68.4)	111 (67.7)
Moderate	46 (29.1)	49 (29.9)
Severe	4 (2.5)	3 (1.8)
Wasting		
Normal	57 (36.1)	78 (47.6)
Mild	86 (54.4)	74 (45.1)
Moderate	13 (8.2)	12 (7.3)
Severe	2 (1.3)	0 (0.0)

Data are presented as mean (SE) for continuous variables and n (%) for categorical variables, unless otherwise stated. ^a^ Anthropometric and nutrient intake data and *P*-values are LSM (SE) from ANOVA. ^b^ Includes partial and exclusive breastfeeding duration

*ANOVA* analysis of variance, *BMI* body mass index, *DC* dietary counseling, *LSM* least squares mean, *MUAC* mid-upper arm circumference, *ONS* oral nutritional supplement, *SE* standard error

### Anthropometric growth

**Fig. 2:**
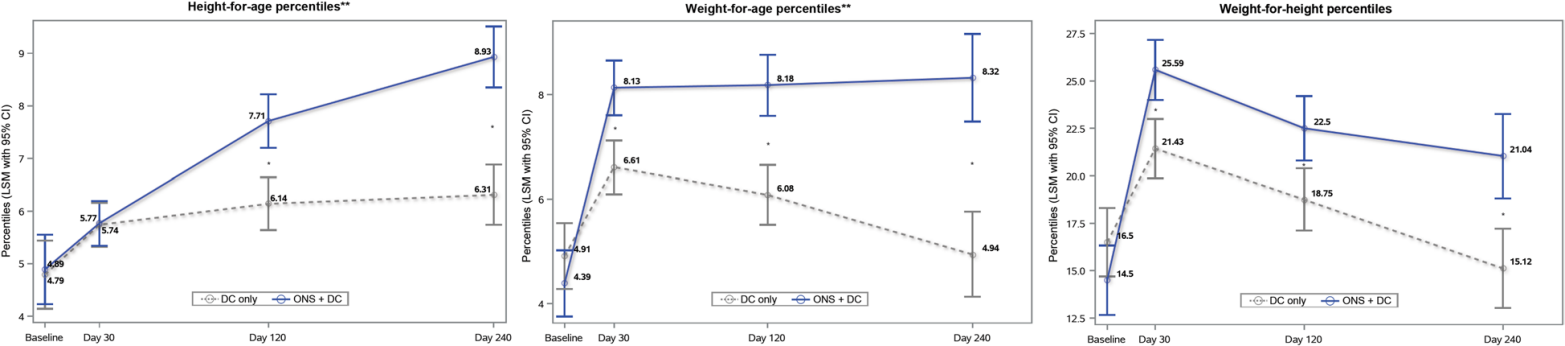
Anthropometric indices at baseline and days 30, 120, and 240 by treatment group. **a** HAP, (**b**) WAP, and (**c**) WHP. Baseline values are ANOVA LSM (SE); days 30, 120, and 240 values are from repeated-measures ANCOVA estimates. *Indicates *P* < 0.05 for between-group comparisons with repeated-measures for that timepoint. **Indicates *P* < 0.05 for the treatment-by-visit interaction effect over the post-baseline visits. ANCOVA, analysis of covariance; ANOVA, analysis of variance; DC, dietary counseling; HAP, height-for-age percentiles; LSM, least squares mean; ONS, oral nutritional supplement; SE, standard error; WAP, weight-for-age percentile; WHP, weight-for-height percentile

The ONS + DC group achieved better weight gain from day 30 and greater height gain from day 120 through day 240 across all weight- and height-for-age indices (Table 2). Between-group differences increased over time, indicated by significant treatment-by-time interactions (Table 2, Fig. 2a–c). The increasing difference in HAP was due to the ONS + DC group’s HAP gains outpacing those of the DC group (Fig. 2a), whereas the widening gap in WAP resulted from a decline in that of the DC group alongside a stable WAP in the ONS + DC group after day 30 (Fig. 2b). Proportional growth, as indicated by WHP, was greater in the supplemented group starting from day 30, although both groups experienced a decline after day 30 (Fig. 2c). The ONS + DC group also had a positive and greater change in height-for-age difference (least squares mean change at day 240 [standard error; SE], cm: 0.70 [0.08] vs –0.03 [0.07]; *P* < 0.001), indicating linear catch-up growth relative to the growth standards [35]. ONS + DC group also had longer lower leg measurements at day 240 (Table 2). Changes in anthropometric indices are available in Supplementary Table S3.

**Table 2:** Anthropometry by group at baseline and days 30, 120, 240

	**Visit**	**ONS + DC**	**DC only**	**Difference**	***P*-value** **(between groups)**	***P*-value** **(treatment-by-visit interaction)**
**Weight (kg)**	Baseline	12.51 (0.07)	12.59 (0.07)	-0.08 (0.09)	0.37	–
	Day 30	13.20 (0.03)	12.99 (0.03)	0.21 (0.05)	** < 0.001**	** < 0.001**
	Day 120	13.57 (0.04)	13.26 (0.04)	0.31 (0.05)	** < 0.001**	
	Day 240	14.03 (0.05)	13.60 (0.05)	0.44 (0.07)	** < 0.001**	
**WAZ**	Baseline	–1.91 (0.05)	–1.85 (0.04)	-0.06 (0.06)	0.37	–
	Day 30	–1.57 (0.02)	–1.69 (0.02)	0.12 (0.03)	** < 0.001**	**0.003**
	Day 120	–1.58 (0.02)	–1.75 (0.02)	0.17 (0.03)	** < 0.001**	
	Day 240	–1.63 (0.03)	–1.86 (0.03)	0.23 (0.04)	** < 0.001**	
**Height (cm)**	Baseline	94.28 (0.18)	94.17 (0.18)	0.11 (0.25)	0.66	–
	Day 30	95.18 (0.06)	95.16 (0.06)	0.02 (0.08)	0.76	** < 0.001**
	Day 120	97.28 (0.07)	96.82 (0.07)	0.46 (0.09)	** < 0.001**	
	Day 240	99.65 (0.07)	98.94 (0.07)	0.71 (0.10)	** < 0.001**	
**HAZ**	Baseline	–1.84 (0.04)	–1.86 (0.04)	0.02 (0.06)	0.73	
	Day 30	–1.78 (0.01)	–1.78 (0.01)	0.004 (0.018)	0.83	** < 0.001**
	Day 120	–1.62 (0.02)	–1.73 (0.02)	0.11 (0.02)	** < 0.001**	
	Day 240	–1.55 (0.02)	–1.71 (0.02)	0.16 (0.02)	** < 0.001**	
**WHZ**	Baseline	–1.23 (0.05)	–1.12 (0.05)	-0.10 (0.07)	0.13	–
	Day 30	–0.78 (0.03)	–0.93 (0.03)	0.16 (0.04)	**0.001**	0.75
	Day 120	–0.90 (0.04)	–1.06 (0.04)	0.16 (0.05)	**0.002**	
	Day 240	–1.02 (0.05)	–1.22 (0.04)	0.20 (0.06)	**0.002**	
**BMIAZ**	Baseline	–1.07 (0.05)	–0.96 (0.05)	-0.11 (0.07)	0.11	–
	Day 30	–0.64 (0.03)	–0.80 (0.03)	0.16 (0.04)	**0.001**	0.78
	Day 120	–0.80 (0.04)	–0.94 (0.04)	0.14 (0.05)	**0.005**	
	Day 240	–0.96 (0.04)	–1.13 (0.04)	0.17 (0.05)	**0.003**	
**MUACZ**	Baseline	–1.23 (0.05)	–1.19 (0.05)	-0.04 (0.07)	0.53	–
	Day 30	–1.17 (0.03)	–1.28 (0.03)	0.11 (0.04)	**0.01**	**0.01**
	Day 120	–1.17 (0.03)	–1.28 (0.03)	0.11 (0.05)	**0.02**	
	Day 240	–0.91 (0.04)	–1.14 (0.04)	0.23 (0.06)	** < 0.001**	
**Lower leg length (cm)**	Baseline	26.42 (0.08)	26.57 (0.08)	-0.15 (0.11)	0.12	
	Day 120	27.62 (0.04)	27.54 (0.04)	0.09 (0.05)	0.09^†^	** < 0.001**
	Day 240	28.75 (0.04)	28.51 (0.04)	0.24 (0.06)	** < 0.001**	

Data are presented as LSM (SE). Baseline values: ANOVA. Anthropometry endpoints: repeated-measures ANCOVA. *P*-values in bold are *P* < 0.05

*ANCOVA* analysis of covariance, *ANOVA* analysis of variance, *BMIAZ* body mass index-for-age z-score, *DC* dietary counseling, *HAZ* height-for-age z-score, *LSM* least squares mean, *MUACZ* mid-upper arm circumference-for-age z-score, *ONS* oral nutritional supplement, *SE* standard error, *WAZ* weight-for-age z-score, *WHZ* weight-for-height z-score

### Dietary intake and nutritional blood biomarkers

Energy and macronutrient intakes were higher in the supplemented group at all post-baseline visits (*P* < 0.001, for all comparisons). At day 240, the supplemented group had 26%, 22%, 20% and 42% higher intake levels of energy, protein, carbohydrate, and fat, respectively, compared to the control group (Table 3). Additionally, more children in the supplemented group achieved adequate energy, carbohydrate, and fat intake levels at all post-baseline visits (*P* < 0.001, for all comparisons). Protein adequacy was high at baseline (88.2% in the overall study population) and greater than 90% at all post-baseline visits in both groups, with the ONS + DC group having a higher proportion of adequacy only at day 120 (Table 3). Median compliance with the study ONS in the supplemented group was very high at 97.7%.

**Table 3:** Energy and macronutrient intake and adequacy at days 30, 120, and 240

	**Visit**	**ONS + DC**	**DC only**	***P*-value**
**Nutrient Intake**^**a**^				
Energy (kcal)	Baseline	835.0 (17.9)	867.60 (17.4)	**-**
	Day 30	1204 (21)	955 (21)	** < 0.001**
	Day 120	1261 (19)	947 (19)	** < 0.001**
	Day 240	1284 (24)	1022 (23)	** < 0.001**
Protein (g)	Baseline	33.0 (1.0)	34.69 (1.0)	**-**
	Day 30	44.9 (1.1)	37.1 (1.0)	** < 0.001**
	Day 120	47.7 (0.9)	36.9 (0.9)	** < 0.001**
	Day 240	48.7 (1.0)	39.8 (1.0)	** < 0.001**
Carbohydrate (g)	Baseline	128.0 (2.7)	130.15 (2.7)	**-**
	Day 30	175.0 (3.3)	143.8 (3.2)	** < 0.001**
	Day 120	180.0 (3.0)	143.3 (3.0)	** < 0.001**
	Day 240	186.0 (3.7)	154.9 (3.6)	** < 0.001**
Fat (g)	Baseline	21.2 (0.9)	23.00 (0.8)	**-**
	Day 30	35.8 (1.1)	25.6 (1.0)	** < 0.001**
	Day 120	38.7 (0.9)	25.1 (0.9)	** < 0.001**
	Day 240	38.2 (1.1)	26.9 (1.0)	** < 0.001**
**Achieving Nutrient Adequacy (%)**^**b**^				
Energy	Baseline	49 (31.0)	58 (35.2)	-
	Day 30	132 (83.5)	80 (49.1)	** < 0.001**
	Day 120	145 (92.9)	73 (45.1)	** < 0.001**
	Day 240	135 (88.2)	91 (56.2)	** < 0.001**
Protein	Baseline	136 (86.1)	149 (90.3)	-
	Day 30	157 (99.4)	157 (96.3)	0.121
	Day 120	156 (100.0)	151 (93.2)	** < 0.001**
	Day 240	150 (98.0)	154 (95.1)	0.220
Carbohydrate	Baseline	48 (30.4)	61 (37.0)	-
	Day 30	129 (81.6)	83 (50.9)	** < 0.001**
	Day 120	135 (86.5)	84 (51.9)	** < 0.001**
	Day 240	133 (86.9)	97 (59.9)	** < 0.001**
Fat	Baseline	24 (15.2)	28 (17.0)	-
	Day 30	87 (55.1)	48 (29.4)	** < 0.001**
	Day 120	108 (69.2)	38 (23.5)	** < 0.001**
	Day 240	99 (64.7)	43 (26.5)	** < 0.001**

^a^Nutrient intake data are presented as LSM (SE). Baseline values: ANCOVA; Day 30/120/240 analyses: Repeated-measures ANCOVA

^b^Nutrient adequacy data are presented as numbers (%) achieving nutrient adequacy based on age- and sex-specific estimated average requirement, derived from Vietnamese RNIs. *P*-values are from chi-squared tests

*P*-values in bold are *P* < 0.05

*ANCOVA*, analysis of covariance, *DC* dietary counseling, *LSM* least squares mean, *ONS* oral nutritional supplement, *SE* standard error

At baseline, 86.6% of the overall study population were vitamin D insufficient (< 30 μg/L) and 25.3% were vitamin D deficient (< 20 μg/L). At day 240, the ONS + DC group had higher serum vitamin D levels and were more likely to achieve sufficiency (≥ 30 ug/L) than the control group (73.5% vs 61.7%; OR [95% CI]: 3.24 [0.96–10.88]; *P* = 0.03) (Table 4). They also had better vitamin K status, indicated by a lower serum undercarboxylated osteocalcin to carboxylated osteocalcin ratio (Table 4). No differences in serum amino acid levels were found between groups at day 240 (data not shown). Very few participants (< 2% of the overall study population) had low hemoglobin or low serum albumin levels at baseline, and no differences in serum concentration or status of these nutritional biomarkers were observed between groups at day 240. At the individual level, all four children with low baseline serum albumin (≤ 3.8 g/d) recovered to normal levels by day 240. Five children had low hemoglobin levels (< 11 g/dL) at baseline, and eight children had low hemoglobin levels (< 11 g/dL) at day 240 (*n* = 6 [ONS + DC], *n* = 2 [DC only]). The difference in hemoglobin status between groups was not statistically significant at day 240 (95.9% vs. 98.8% with normal hemoglobin ≥ 11 g/dL; *p* = 0.12). Supplementary Table S4 provides individual-level information of participants with low hemoglobin levels at any time point.

**Table 4:** Nutritional blood biomarkers concentration and status

	**Visits**	**ONS + DC**	**DC only**		***P*-value**
**Serum concentration**		**Mean**		**Difference**	
Total 25-hydroxy vitamin D (µg/L)	Baseline	24.06 (0.50)	23.78 (0.49)	0.28 (0.67)	–
	Day 240	34.25 (0.61)	32.30 (0.60)	1.95 (0.79)	**0.01**
ucOC (ng/mL)	Baseline	40.19 (2.24)	38.67 (2.20)	1.52 (3.00)	–
	Day 240	34.46 (2.58	45.41 (2.54)	–10.95 (3.33)	**0.001**
cOC (ng/mL)	Baseline	31.26 (0.70)	30.42 (0.69)	0.84 (0.93)	–
	Day 240	28.95 (0.83)	31.57 (0.81)	–2.62 (1.07)	**0.02**
ucOC:cOC ratio	Baseline	1.27 (0.06)	1.27 (0.06)	0.01 (0.09)	–
	Day 240	1.22 (0.08)	1.48 (0.08)	–0.27 (0.10)	**0.01**
Albumin (g/dL)	Baseline	4.40 (0.02)	4.39 (0.02)	0.01 (0.03)	–
	Day 240	4.61 (0.05)	4.59 (0.05)	0.02 (0.06)	0.72
Hemoglobin (g/dL)	Baseline	13.03 (0.07)	12.99 (0.07)	0.04 (0.10)	–
	Day 240	12.96 (0.05)	12.93 (0.05)	0.03 (0.07)	0.68
**Nutritional blood biomarker status**		**Numbers (%)**		**Odds ratio (95% CI)**	
Hemoglobin ≥ 11 g/dL	Baseline	145 (98.0)	158 (98.7)	–	–
	Day 240	141 (95.9)	158 (98.8)	–	0.12^a^
Vitamin D ≥ 30 ug/L (sufficient)	Baseline	16 (11.8)	21 (14.9)	–	**–**
	Day 240	100 (73.5)	87 (61.7)	3.24 (0.96 – 10.88)	**0.03**^b^
Vitamin D ≥ 20 ug/L (not deficient)	Baseline	98 (72.1)	109 (77.3)	–	–
	Day 240	135 (99.3)	136 (96.5)	–	0.21^a^
Albumin > 3.8 g/dL	Baseline	134 (98.5)	139 (98.6)	–	–
	Day 240	136 (100.0)	141 (100.0)	NA	NA

Data are presented as LSM (SE) for serum concentrations and as numbers (%) in each group achieving the cutoffs for nutritional status classification. Baseline values for serum concentration: ANOVA. Endpoint values: ANCOVA. *P*-values in bold are *P* < 0.05

^a^*P*-values are from Fisher’s exact test due to the small number of observations in each cell

^b^*P*-value is from a binomial logistic regression model

*ANCOVA* analysis of covariance, *ANOVA* analysis of variance, *cOC* carboxylated osteocalcin, *DC* dietary counseling, *LSM* least squares mean, *ONS* oral nutritional supplement, *NA* Not analyzed, *SE* standard error, *ucOCC* undercarboxylated osteocalcin

### Body composition and bone mineral content and density

**Fig. 3:**
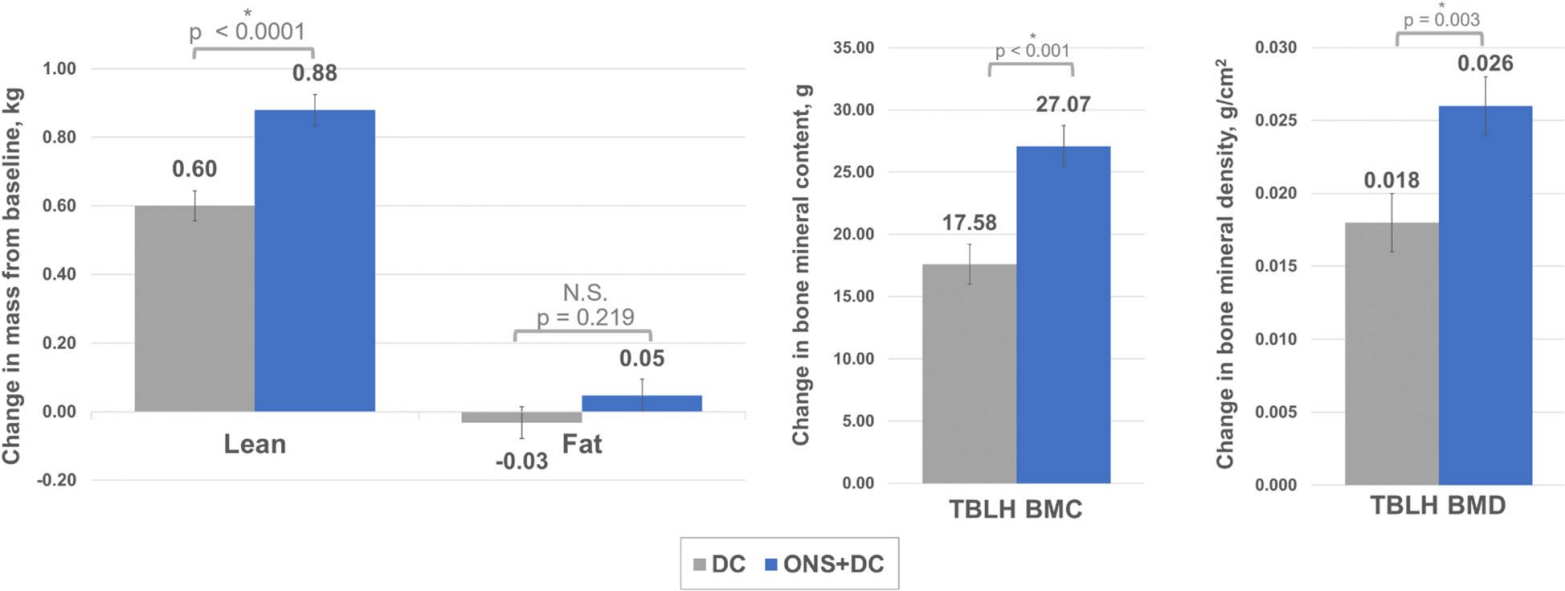
Change in DXA-assessed measurements from baseline to day 240 by treatment group. **a** Lean and fat mass, (**b**) bone mineral content, and (**c**) bone mineral density. Values are LSM (SE) of changes from baseline to day 240 from ANCOVA estimates. *Indicates *P* < 0.05 for between-group comparisons. ANCOVA, analysis of covariance; BMC, bone mineral content; BMD, bone mineral density; DC, dietary counseling; DXA, dual-energy X-ray absorptiometry; LSM, least squares mean; ONS, oral nutritional supplement; SE, standard error; TBLH, total body less head

ONS + DC group gained more total body lean mass (change, kg: 0.88 [0.045] vs 0.60 [0.044]; *P* < 0.001), whereas fat mass gain was not different between groups (*P* = 0.22) at day 240 (Fig. 3a). Height-adjusted lean mass index was also higher in the ONS + DC group (kg/m^2^: 11.06 [0.05] vs 10.92 [0.05]; *P* = 0.048), indicating that the lean mass increase was more than proportional to the greater height gain (Table 5). No differences in handgrip strength were observed between groups at any time point. Total body less head bone mineral content (BMC) and BMD, change, and percentage change were larger in the ONS + DC group than the control group at day 240 (BMC, g: 270.6 [1.8] vs 259.6 [1.7]; *P* < 0.001; and BMD, g/cm^2^: 0.407 [0.003] vs 0.399 [0.003]; *P* = 0.03) (Table 6, Fig. 3b–c). Higher BMC and BMD were also observed in the ONS + DC group at the bilateral total hip and spine. However, mean differences in BMC of the left hip, as well as spine BMD and BMAD, were higher but did not reach statistical significance (Table 6).

**Table 5:** Body composition by group at baseline and day 240

	**Visit**	**ONS + DC**	**DC only**	**Difference**	***P*-value** **(between groups)**
**Lean mass (kg)**	Baseline	10.104 (0.061)	10.127 (0.061)	-0.023 (0.008)	0.78
	Day 240	10.970 (0.050)	10.800 (0.049)	0.170 (0.069)	**0.01**
**Lean mass index (kg/m**^**2**^**)**	Baseline	11.309 (0.073)	11.370 (0.073)	-0.062 (0.099)	0.53
	Day 240	11.055 (0.051)	10.918 (0.049)	0.137 (0.069)	**0.05**
**Fat mass (kg)**	Baseline	2.102 (0.043)	2.090 (0.043)	0.013 (0.058)	0.82
	Day 240	2.151 (0.048)	2.081 (0.047)	0.070 (0.066)	0.29
**Fat mass index (kg/m**^**2**^**)**	Baseline	2.396 (0.050)	2.362 (0.049)	0.034 (0.067)	0.62
	Day 240	2.183 (0.050)	2.135 (0.048)	0.048 (0.067)	0.48

Data are presented as LSM (SE). Baseline values: ANOVA. Day 240 values: ANCOVA. *P*-values in bold are *P* < 0.05

*ANCOVA* analysis of covariance, *ANOVA* analysis of variance, *DC* dietary counseling, *LSM* least squares mean, *ONS* oral nutritional supplement, *SE* standard error

**Table 6:** Bone mineral content and density measurements, changes at baseline and day 240

		**ONS + DC**	**DC only**	**Difference**	***P*-value**
**TBLH**					
** BMC (g)**	Baseline	239.48 (1.77)	241.02 (1.73)	–1.55 (2.32)	0.51
	Day 240	270.59 (1.75)	259.58 (1.67)	11.00 (2.31)	** < 0.001**
	Change	27.07 (1.67)	17.58 (1.61)	9.50 (2.23)	** < 0.001**
	% Change	11.402 (0.684)	8.062 (0.659)	3.340 (0.914)	** < 0.001**
** BMD (g/cm**^**2**^**)**	Baseline	0.380 (0.004)	0.384 (0.004)	–0.005 (0.005)	0.35
	Day 240	0.407 (0.003)	0.399 (0.003)	0.008 (0.003)	**0.03**
	Change	0.026 (0.002)	0.018 (0.002)	0.008 (0.003)	**0.003**
	% Change	6.839 (0.535)	5.014 (0.517)	1.825 (0.716)	**0.01**
**Right hip, total**					
** BMC (g)**	Baseline	7.454 (0.075)	7.539 (0.074)	–0.085 (0.101)	0.40
	Day 240	8.576 (0.069)	8.351 (0.067)	0.225 (0.092)	**0.02**
	Change	1.036 (0.069)	0.811 (0.067)	0.225 (0.092)	**0.02**
	% Change	16.397 (1.587)	11.774 (1.547)	4.624 (2.119)	**0.03**
** BMD (g/cm**^**2**^**)**	Baseline	0.523 (0.005)	0.522 (0.005)	0.000 (0.006)	0.99
	Day 240	0.565 (0.004)	0.552 (0.003)	0.013 (0.005)	**0.007**
	Change	0.038 (0.004)	0.025 (0.003)	0.013 (0.005)	**0.007**
	% Change	7.663 (0.659)	5.330 (0.642)	2.333 (0.877)	**0.008**
**Left hip, total**					
** BMC (g)**	Baseline	7.499 (0.084)	7.448 (0.083)	0.051 (0.113)	0.65
	Day 240	8.442 (0.086)	8.264 (0.084)	0.178 (0.115)	0.12
	Change	0.958 (0.086)	0.780 (0.084)	0.178 (0.115)	0.12
	% change	13.010 (1.398)	12.341 (1.359)	0.669 (1.862)	0.72
** BMD (g/cm**^**2**^**)**	Baseline	0.518 (0.004)	0.517 (0.004)	0.001 (0.006)	0.88
	Day 240	0.561 (0.003)	0.550 (0.003)	0.011 (0.005)	**0.02**
	Change	0.040 (0.003)	0.029 (0.003)	0.011 (0.005)	**0.02**
	% Change	7.802 (0.65)	6.077 (0.632)	1.725 (0.866)	**0.05**
**Spine (L1–L4)**					
** BMC (g)**	Baseline	10.232 (0.106)	10.129 (0.105)	0.103 (0.143)	0.47
	Day 240	11.757 (0.100)	11.418 (0.097)	0.339 (0.133)	**0.01**
	Change	1.539 (0.100)	1.199 (0.097)	0.339 (0.133)	**0.01**
	% change	15.653 (1.134)	12.730 (1.110)	2.923 (1.516)	0.06^†^
** BMD (g/cm**^**2**^**)**	Baseline	0.433 (0.004)	0.434 (0.004)	–0.001 (0.005)	0.88
	Day 240	0.463 (0.004)	0.454 (0.004)	0.009 (0.005)	0.07^†^
	Change	0.029 (0.004)	0.020 (0.004)	0.009 (0.005)	0.07^†^
	% Change	6.898 (1.027	5.526 (1.004)	1.372 (1.372)	0.32
** BMAD (g/cm**^**3**^**)**	Baseline	0.177 (0.002)	0.179 (0.002)	–0.002 (0.002)	0.39
	Day 240	0.184 (0.002)	0.180 (0.002)	0.004 (0.002)	0.10
	Change	0.005 (0.002)	0.002 (0.002)	0.004 (0.002)	0.10
	% Change	3.825 (0.999)	1.917 (0.976)	1.908 (1.336)	0.15

Data are presented as LSM (SE). Baseline values: ANOVA. Endpoint values: ANCOVA. *P*-values in bold are *P* < 0.05. ^†^*P*-values are *P* < 0.10 and > 0.05

*ANCOVA* analysis of covariance, *ANOVA* analysis of variance, *BMAD* bone mineral apparent density, *BMC* bone mineral content, *BMD* bone mineral density, *DC* dietary counseling, *LSM* least squares mean, *ONS* oral nutritional supplement, *SE* standard error, *TBLH* total body less head

### Parent-reported child outcomes

A larger proportion of the ONS + DC group did not have any illness episodes during the 8-month study, did not incur healthcare expenditures, and majority of their parents did not miss work due to the child’s illness (Table 7). The ONS + DC group also experienced 37% fewer sick days (*P* = 0.03) and 37% fewer missed school days (*P* = 0.03) than the control group (Table 7). The ONS + DC group had higher parent-reported appetite, physical activity, and energy levels (10-point visual analog scale) (Supplementary Figure S1A–C), shorter night awakenings, a longer night sleep duration, and better sleep quality scores (10-point visual analog scale) across follow-up visits (*P* < 0.05 for the overall treatment effect across follow-up visits in all domains) than the control group (Supplementary Figure S2A–D). No differences in attentional focus between groups were found at day 240 (data not shown). Details of parent-reported outcomes are reported in Supplementary Table S5.

**Table 7:** Parent-reported illness-related outcomes over the 240-day study duration

	**ONS + DC** **(*n* = 163)**	**DC only** **(*n* = 153)**	**Incident rate ratio**	***P*-value**
**Number (percentage) of children in each group during the 240-day study**^a^				
No illness episodes	47 (31)	32 (20)	1.16 (1.02 – 1.32)	**0.03**
No missed school days	87 (57)	75 (46)	–	^†^0.05
No missed work days (parent)	136 (89)	130 (80)	1.89 (1.08 – 3.32)	**0.02**
No healthcare expenditures	53 (35)	37 (23)	1.18 (1.03 – 1.37)	**0.02**
**Number of episodes or days during the 240-day study**^b^				
Frequency of acute illnesses	1.4	1.6	0.90 (0.74 – 1.08)	0.25
Number of sick days	2.4	3.8	0.63 (0.41 – 0.96)	**0.03**
Number of missed school days	2.3	3.6	0.63 (0.41 – 0.96)	**0.03**
Number of missed work days (parent)	0.5	0.9	0.57 (0.23 – 1.37)	0.21

Data are presented as numbers (percentages) in each group unless otherwise stated. *P*-values in bold are *P* < 0.05. ^†^*P*-values ≥ 0.05 to < 0.10

^a^Incident rate ratios and *P*-values are from Poisson regression models

^b^Numbers of episodes or days are exponentiated LSM, incident rate ratios and *P*-values are from negative binomial regression models

*DC* dietary counseling, *LSM* least squares mean, *ONS* oral nutritional supplement

### Adverse events

A total of 71% of participants in the ONS + DC group and 81% in the control DC-only group reported at least one adverse event (AE) during the study period. The majority of AEs reported were mild (95%) and deemed not related or probably not related to the study product (99%). AEs commonly associated with gastrointestinal tolerance (i.e. constipation, diarrhea, and vomiting) were reported in 4% of participants, with no significant differences observed between the ONS + DC and DC-only groups. The majority of AEs (98%) were nonserious, with 11 participants (3%) reporting a serious AE (SAE). This included one participant from the ONS + DC group and 10 participants from the DC-only group. None of the SAEs were related to the study interventions. Overall, the study ONS was found to be safe and well tolerated.

## Discussion

Long-term (8-months) ONS + DC significantly improved height, weight, and proportionate weight; increased lean mass accretion and bone mineralization; enhanced nutrient intake and adequacy, and vitamins K and D status; and improved parent-reported immunity-related and other health outcomes in children aged 2–5-years old with or at risk of undernutrition, when compared to a DC-only strategy. Notably, ONS supported quality growth evidenced by linear catch-up growth alongside a higher lean mass index and BMD, which indicate that the greater lean and bone mass accreted contributed to both height gain and improved composition, without excessive weight or fat mass gain.

To the best of our knowledge, SPROUT is the longest RCT of ONS in children aged 2 to 5 years with or at risk of undernutrition. The pattern of continuous HAP improvement alongside WAP stabilization after day 30 in SPROUT is similar to a previous single-arm, 48-week study of ONS + DC in Filipino children with or at risk of undernutrition [25]. In SPROUT, comparison with the control group isolates the incremental effects of ONS from DC. The growing differences in HAP and WAP between groups highlight the cumulative impact of incremental growth over time. Beyond physical size, SPROUT is the first ONS RCT to investigate growth quality in terms of lean and bone mass accretion, showing approximately 50% more accretion of lean mass and BMC in the supplemented group compared to the control group. It is also the first RCT of supplementary foods to show improved linear growth alongside improvement of lean mass index and BMD. Earlier trials of supplementary foods assessing BC have used two-component methods, such as deuterium dilution or bioelectrical impedance analysis, and therefore did not differentiate fat-free mass into lean and bone mass. Our findings of lean/fat-free mass gain without significant fat mass gain are broadly in line with recent shorter-term, 4- to 16-week trials of supplementary foods fortified with a wide range of micronutrients in moderate-to-severe undernutrition in children [18, 36–38]. However, SPROUT is unique due to its longer duration and inclusion of children with mild undernutrition. Apart from total body BMC and BMD, BMC and BMD were also higher at the spine and bilateral total hip, though a few parameters did not reach statistical significance. This could be due to varying DXA measurement precision across sites [39], varying bone acquisition rates [39–41], or potential site selectivity of nutrients [42]. Indeed, the minimum monitoring time interval, which is the time needed to identify a change between two scans that exceeds measurement error, is shortest for total body less head, followed by the spine and hip sites for BMD in children aged 6 years [39]. Thus, longer trials may be needed to demonstrate effects at these sites conclusively.

Holistic improvement in child health beyond physical growth was also observed in terms of fewer parent-reported sick days; increased appetite, physical activity, and energy levels; and better sleep habits in the ONS + DC group. These effects were seen at day 120 and discussed extensively earlier [26]. Briefly, apart from supporting lean tissue synthesis, energy, and growth nutrients such as iron and zinc may contribute to increased physical activity, appetite, and sleep in undernourished children [43–46]. Adequate protein and micronutrient intake also support immune function [47]. Supplementation increased nutrient intake and helped to close the adequacy gap, with only 12%, 13%, and 35% of the ONS + DC group not achieving adequate energy, carbohydrate, and fat intake at day 240, compared to 44%, 40%, and 73%, respectively, in the DC-only group. Two servings of study ONS provided a balanced ratio of macronutrients, at least 30% of the recommended daily allowance for all nutrients, and other nutrients that support growth and bone health, including arginine, vitamin K_2_, and casein phosphopeptide. Higher arginine intake and serum arginine are associated with better height gain and status in children [48, 49]. Vitamin K_2_ is a co-factor needed to carboxylate osteocalcin, which acts as a calcium chaperone that can improve bone mineralization [50, 51]. Casein phosphopeptide chelates key minerals and helps maintain them in a soluble state [52–54], which may increase absorption of calcium and zinc.

Formulas containing a broad spectrum of nutrients are likely more effective than single or selected micronutrient supplements, as multiple nutrient deficiencies often coexist in poor growth [55]. Improvements in one health domain may also indirectly benefit other health domains. For example, while better vitamin D and vitamin K status may be directly associated with improved BM in undernourished children [56, 57], higher physical activity and greater lean mass, which may be attributed to energy, protein, and zinc intakes, are also factors that contribute indirectly to improved BM [58, 59]. Many outcomes in the SPROUT study are interrelated, including immunity and growth [47]; appetite, nutrient intake, and physical activity [43, 44]; physical activity, sleep, lean mass, and bone health [58, 60, 61]; and sleep and growth [62]. Future analyses will be conducted to identify associations and pathways of these improvements.

The key strengths of the SPROUT study are its high study completion and compliance rates contributing to study validity, and the use of DXA, a widely accepted and well-validated method of BM and BC assessment [63–65]. The 8-month duration provides long-term efficacy and safety data. AEs reported were typical in nature for the population, and the rates of gastrointestinal AEs were comparable in type and incidence to other studies of ONS in undernourished children [66].

Several study limitations should be noted. First, reference data that is age-, sex- and ethnicity- specific for the computation of standardized scores for BC, BMC and BMD relative to the general population are lacking. While absolute improvements in the ONS + DC group demonstrate improved lean mass and bone mass accretion compared to the control group, the lack of relative indices (such as z-scores or percentiles) limits interpretation of whether the intervention supported adequate gain for improvements relative to the general population, including normally-nourished children. Another limitation is the study’s open-label design, where participants, parents and investigators were aware of treatment assignment, introducing potential bias. Parent-reported outcomes, including illness frequency, sleep habits, energy, physical activity and appetite levels are particularly susceptible to reporting biases, compared to objective measures such as anthropometry where assessors were blinded, DXA scans and nutritional blood biomarkers. Using objective assessment methods such as medical records review, sleep actigraphy, or activity trackers in future studies can mitigate such biases and confirm these findings. Third, as children were enrolled from the community, very few severely undernourished children were included in this study, limiting generalizability to such children. Finally, the fixed dose of ONS used across age groups, rather than individualized or an adjustable weight-based dosing, limits the study’s ability to measure efficacy of an optimized ONS dose that may be used in practice.

## Conclusion

Long-term ONS + DC for 8 months improves linear catch-up growth and growth quality, as evidenced by greater lean and bone mass accretion, compared to DC alone. In this study of mostly mild-to-moderately undernourished children, the nutrient blend in ONS supports height gain, the accretion of lean soft and skeletal tissue without excessive weight or fat gain, and improved serum nutritional status of vitamin D and K. Other additional parent-reported outcomes in immunity, appetite, sleep, physical activity, and energy levels suggest holistic improvement in multiple domains of child health and well-being. Further research should aim to confirm these additional benefits through objective assessment methods, and to investigate the mechanisms by which ONS influences growth and health improvements.

## Supplementary Information

Supplementary Material 1: Supplementary Tables S1-5, Supplementary Figures S1-2. Supplementary File S1.

## Data Availability

Ethical restrictions imposed by the Institutional Review Board prevent public sharing of data for this study in children. The data used in this publication is owned by Abbott Nutrition. All authors have ongoing access to study data. Data access requests will be evaluated by Abbott Nutrition in consideration of Institutional Review Board requirements. Interested researchers will need to sign a research collaboration agreement with Abbott. Requests can be sent to mandyyenling.ow@abbott.com.

## Abbreviations

AE: Adverse event
ANCOVA: Analysis of covariance
BC: Body composition
BM: Bone mineralization
BMAD: Bone mineral apparent density
BMC: Bone mineral content
BMD: Bone mineral density
BMIAZ: Body mass index-for-age z-score
cOC: Carboxylated osteocalcin
CONSORT: Consolidated Standards of Reporting Trials
DC: Dietary counseling
DXA: Dual-energy X-ray absorptiometry
HAP: Height-for-age percentile
HAZ: Height-for-age z-score
ITT: Intent-to-treat
LSM: Least squares mean
MUAC: Mid-upper arm circumference
MUACZ: Mid-upper arm circumference-for-age z-score
ONS: Oral nutritional supplements
RCT: Randomized controlled trial
RNI: Recommended nutrient intake
SE: Standard error
SPROUT: Supporting Pediatric GRowth and Health OUTcomes
TBLH: Total body less head
ucOC: Undercarboxylated osteocalcin
WAP: Weight-for-age percentile
WAZ: Weight-for-age z-score
WHO: World Health Organization
WHP: Weight-for-height percentile
WHZ: Weight-for-height z-score

## References

[1] Black RE, Allen LH, Bhutta ZA, Caulfield LE, de Onis M, Ezzati M, Mathers C, Rivera J. Maternal and child undernutrition: global and regional exposures and health consequences. Lancet. 2008;371:243–60. 18207566 10.1016/S0140-6736(07)61690-0

[2] De Sanctis V, Soliman A, Alaaraj N, Ahmed S, Alyafei F, Hamed N. Early and Long-term Consequences of Nutritional Stunting: From Childhood to Adulthood. Acta Biomed. 2021;92:e2021168. 33682846 10.23750/abm.v92i1.11346 PMC7975963

[3] Wells JCK. Body composition of children with moderate and severe undernutrition and after treatment: a narrative review. BMC Med. 2019;17:215. 31767002 10.1186/s12916-019-1465-8 PMC6878632

[4] Ekbote VH, Khadilkar AV, Chiplonkar SA, Khadilkar VV. Determinants of bone mineral content and bone area in Indian preschool children. J Bone Miner Metab. 2011;29:334–41. 20941516 10.1007/s00774-010-0224-x

[5] Ferrer FS, Castell EC, Marco FC, Ruiz MJ, Rico JAQ, Roca APN. Influence of weight status on bone mineral content measured by DXA in children. BMC Pediatr. 2021;21:185. 33879114 10.1186/s12887-021-02665-5 PMC8056645

[6] Martins VJ, Toledo Florencio TM, Grillo LP. do Carmo PFM, Martins PA, Clemente AP, Santos CD, de Fatima AVM, Sawaya AL: Long-lasting effects of undernutrition. Int J Environ Res Public Health. 2011;8:1817–46. 21776204 10.3390/ijerph8061817 PMC3137999

[7] Ambroszkiewicz J, Gajewska J, Rowicka G, Klemarczyk W, Chelchowska M. Assessment of Biochemical Bone Turnover Markers and Bone Mineral Density in Thin and Normal-Weight Children. Cartilage. 2018;9:255–62. 29156943 10.1177/1947603516686145 PMC6042038

[8] Hron BM, Duggan CP. Pediatric undernutrition defined by body composition-are we there yet? Am J Clin Nutr. 2020;112:1424–6. 33094806 10.1093/ajcn/nqaa292 PMC7727470

[9] Lara-Pompa NE, Hill S, Williams J, Macdonald S, Fawbert K, Valente J, Kennedy K, Shaw V, Wells JC, Fewtrell M. Use of standardized body composition measurements and malnutrition screening tools to detect malnutrition risk and predict clinical outcomes in children with chronic conditions. Am J Clin Nutr. 2020;112:1456–67. 32520318 10.1093/ajcn/nqaa142

[10] Wells JCK. Double burden of malnutrition in thin children and adolescents: low weight does not protect against cardiometabolic risk. Eur J Clin Nutr. 2021;75:1167–9. 34230630 10.1038/s41430-021-00963-w PMC8352780

[11] Palacios-Marin I, Serra D, Jiménez-Chillarón JC, Herrero L, Todorčević M. Childhood obesity: Implications on adipose tissue dynamics and metabolic health. Obes Rev. 2023;24:e13627. 37608466 10.1111/obr.13627

[12] Singhal A. Long-Term Adverse Effects of Early Growth Acceleration or Catch-Up Growth. Ann Nutr Metab. 2017;70:236–40. 28301849 10.1159/000464302

[13] Clark EM, Ness AR, Bishop NJ, Tobias JH. Association between bone mass and fractures in children: a prospective cohort study. J Bone Miner Res. 2006;21:1489–95. 16939408 10.1359/jbmr.060601 PMC2742714

[14] Wren TA, Kalkwarf HJ, Zemel BS, Lappe JM, Oberfield S, Shepherd JA, Winer KK, Gilsanz V. Longitudinal tracking of dual-energy X-ray absorptiometry bone measures over 6 years in children and adolescents: persistence of low bone mass to maturity. J Pediatr. 2014;164:1280-1285.e1282. 24485819 10.1016/j.jpeds.2013.12.040 PMC4035430

[15] Kalkwarf HJ, Gilsanz V, Lappe JM, Oberfield S, Shepherd JA, Hangartner TN, Huang X, Frederick MM, Winer KK, Zemel BS. Tracking of bone mass and density during childhood and adolescence. J Clin Endocrinol Metab. 2010;95:1690–8. 20194709 10.1210/jc.2009-2319 PMC2853985

[16] Weaver CM, Gordon CM, Janz KF, Kalkwarf HJ, Lappe JM, Lewis R, O’Karma M, Wallace TC, Zemel BS. The National Osteoporosis Foundation’s position statement on peak bone mass development and lifestyle factors: a systematic review and implementation recommendations. Osteoporos Int. 2016;27:1281–386. 26856587 10.1007/s00198-015-3440-3 PMC4791473

[17] Golden MH. Proposed recommended nutrient densities for moderately malnourished children. Food Nutr Bull. 2009;30:S267–342. 19998863 10.1177/15648265090303S302

[18] Fabiansen C, Yameogo CW, Iuel-Brockdorf AS, Cichon B, Rytter MJH, Kurpad A, Wells JC, Ritz C, Ashorn P, Filteau S, et al. Effectiveness of food supplements in increasing fat-free tissue accretion in children with moderate acute malnutrition: A randomised 2 x 2 x 3 factorial trial in Burkina Faso. PLoS Med. 2017;14:e1002387. 28892496 10.1371/journal.pmed.1002387 PMC5593178

[19] Alarcon PA, Lin LH, Noche M Jr, Hernandez VC, Cimafranca L, Lam W, Comer GM. Effect of oral supplementation on catch-up growth in picky eaters. Clin Pediatr (Phila). 2003;42:209–17. 12739919 10.1177/000992280304200304

[20] Ghosh AK, Kishore B, Shaikh I, Satyavrat V, Kumar A, Shah T, Pote P, Shinde S, Berde Y, Low YL, et al. Effect of oral nutritional supplementation on growth and recurrent upper respiratory tract infections in picky eating children at nutritional risk: a randomized, controlled trial. J Int Med Res. 2018;46:2186–201. 29614897 10.1177/0300060518757355 PMC6023057

[21] Khanna D, Yalawar M, Saibaba PV, Bhatnagar S, Ghosh A, Jog P, Khadilkar AV, Kishore B, Paruchuri AK, Pote PD, et al. Oral Nutritional Supplementation Improves Growth in Children at Malnutrition Risk and with Picky Eating Behaviors. Nutrients. 2021;13:3590. 34684591 10.3390/nu13103590 PMC8538528

[22] Lebenthal Y, Yackobovitch-Gavan M, Lazar L, Shalitin S, Tenenbaum A, Shamir R, Phillip M. Effect of a nutritional supplement on growth in short and lean prepubertal children: a prospective, randomized, double-blind, placebo-controlled study. J Pediatr. 2014;165(1190–1193):e1191. 10.1016/j.jpeds.2014.08.011 25241181

[23] Pham DT, Hoang TN, Ngo NT, Nguyen LH, Tran TQ, Pham HM, Huynh DTT, Ninh NT. Effect of Oral Nutritional Supplementation on Growth in Vietnamese Children with Stunting. Open Nutr J. 2019;13:43–52.

[24] Sheng X, Tong M, Zhao D, Leung TF, Zhang F, Hays NP, Ge J, Ho WM, Northington R, Terry DL, Yao M. Randomized controlled trial to compare growth parameters and nutrient adequacy in children with picky eating behaviors who received nutritional counseling with or without an oral nutritional supplement. Nutr Metab Insights. 2014;7:85–94. 25342910 10.4137/NMI.S15097 PMC4196879

[25] Huynh D, Estorninos E, Capeding R, Oliver J, Low Y, Rosales F. Longitudinal growth and health outcomes in nutritionally at-risk children who received long-term nutritional intervention. J Hum Nutr Diet. 2015;28:623–35. 25808062 10.1111/jhn.12306 PMC6680231

[26] Ow MY, Tran NT, Berde Y, Nguyen TS, Tran VK, Jablonka MJ, Baggs G, Huynh DT. Oral nutritional supplementation with dietary counseling improves catch-up growth and health outcomes in children with or at risk of undernutrition: a randomized controlled trial. Front Nutr. 2024;11:1341963. 39050140 10.3389/fnut.2024.1341963 PMC11266289

[27] World Health Organization: WHO child growth standards: length/height-for-age, weight-for-age, weight-for-length, weight-for-height and body mass index-for-age: methods and development. Geneva: World Health Organization; 2006.

[28] Schulz KF, Grimes DA. Blinding in randomised trials: hiding who got what. Lancet. 2002;359:696–700. 11879884 10.1016/S0140-6736(02)07816-9

[29] National Institute of Nutrition (Vietnam): 10 tips on proper nutrition for period 2013–2020. Hanoi: Medical Publishing House; 2013.

[30] National Institute of Nutrition (Vietnam): The Vietnamese Food Pyramid. Hanoi: Medical Publishing House; 2010.

[31] Gibson RS. Principles of nutritional assessment. USA: Oxford University Press; 2005.

[32] National Institute of Nutrition (Vietnam): Recommended dietary allowances for Vietnamese. Hanoi: Medical Publishing House; 2016.

[33] Prentice A, Parsons TJ, Cole TJ. Uncritical use of bone mineral density in absorptiometry may lead to size-related artifacts in the identification of bone mineral determinants. Am J Clin Nutr. 1994;60:837–42. 7985621 10.1093/ajcn/60.6.837

[34] Kindler JM, Lappe JM, Gilsanz V, Oberfield S, Shepherd JA, Kelly A, Winer KK, Kalkwarf HJ, Zemel BS. Lumbar Spine Bone Mineral Apparent Density in Children: Results From the Bone Mineral Density in Childhood Study. J Clin Endocrinol Metab. 2019;104:1283–92. 30265344 10.1210/jc.2018-01693 PMC6397436

[35] Frongillo EA, Leroy JL, Lapping K. Appropriate Use of Linear Growth Measures to Assess Impact of Interventions on Child Development and Catch-Up Growth. Adv Nutr. 2019;10:372–9. 30805630 10.1093/advances/nmy093 PMC6520037

[36] Mbabazi J, Pesu H, Mutumba R, Filteau S, Lewis JI, Wells JC, Olsen MF, Briend A, Michaelsen KF, Molgaard C, et al. Effect of milk protein and whey permeate in large quantity lipid-based nutrient supplement on linear growth and body composition among stunted children: A randomized 2 x 2 factorial trial in Uganda. PLoS Med. 2023;20:e1004227. 37220111 10.1371/journal.pmed.1004227 PMC10204948

[37] Suri DJ, Potani I, Singh A, Griswold S, Wong WW, Langlois B, Shen Y, Chui KHK, Rosenberg IH, Webb P, Rogers BL. Body Composition Changes in Children during Treatment for Moderate Acute Malnutrition: Findings from a 4-Arm Cluster-Randomized Trial in Sierra Leone. J Nutr. 2021;151:2043–50. 33880554 10.1093/jn/nxab080 PMC8245884

[38] Kangas ST, Kaestel P, Salpeteur C, Nikiema V, Talley L, Briend A, Ritz C, Friis H, Wells JC. Body composition during outpatient treatment of severe acute malnutrition: Results from a randomised trial testing different doses of ready-to-use therapeutic foods. Clin Nutr. 2020;39:3426–33. 32184026 10.1016/j.clnu.2020.02.038 PMC11346517

[39] Shepherd JA, Wang L, Fan B, Gilsanz V, Kalkwarf HJ, Lappe J, Lu Y, Hangartner T, Zemel BS, Fredrick M, et al. Optimal monitoring time interval between DXA measures in children. J Bone Miner Res. 2011;26:2745–52. 21773995 10.1002/jbmr.473 PMC3200454

[40] Bass S, Delmas PD, Pearce G, Hendrich E, Tabensky A, Seeman E. The differing tempo of growth in bone size, mass, and density in girls is region-specific. J Clin Invest. 1999;104:795–804. 10491415 10.1172/JCI7060 PMC408435

[41] Bradney M, Karlsson MK, Duan Y, Stuckey S, Bass S, Seeman E. Heterogeneity in the growth of the axial and appendicular skeleton in boys: implications for the pathogenesis of bone fragility in men. J Bone Miner Res. 2000;15:1871–8. 11028438 10.1359/jbmr.2000.15.10.1871

[42] Chevalley T, Bonjour JP, Ferrari S, Hans D, Rizzoli R. Skeletal site selectivity in the effects of calcium supplementation on areal bone mineral density gain: a randomized, double-blind, placebo-controlled trial in prepubertal boys. J Clin Endocrinol Metab. 2005;90:3342–9. 15755866 10.1210/jc.2004-1455

[43] Aburto NJ, Ramirez-Zea M, Neufeld LM, Flores-Ayala R. The effect of nutritional supplementation on physical activity and exploratory behavior of Mexican infants aged 8–12 months. Eur J Clin Nutr. 2010;64:644–51. 20354559 10.1038/ejcn.2010.52

[44] Naila NN, Mahfuz M, Hossain M, Arndt M, Walson JL, Nahar B, Ahmed T. Improvement in appetite among stunted children receiving nutritional intervention in Bangladesh: results from a com-munity-based study. Eur J Clin Nutr. 2021;75:1359–67. 34045689 10.1038/s41430-020-00843-9 PMC8416653

[45] Ji X, Grandner MA, Liu J. The relationship between micronutrient status and sleep patterns: a systematic review. Public Health Nutr. 2017;20:687–701. 27702409 10.1017/S1368980016002603 PMC5675071

[46] Kordas K, Siegel EH, Olney DK, Katz J, Tielsch JM, Kariger PK, Khalfan SS, LeClerq SC, Khatry SK, Stoltzfus RJ. The effects of iron and/or zinc supplementation on maternal reports of sleep in infants from Nepal and Zanzibar. J Dev Behav Pediatr. 2009;30:131–9. 19322104 10.1097/DBP.0b013e31819e6a48 PMC2771202

[47] Ibrahim MK, Zambruni M, Melby CL, Melby PC. Impact of Childhood Malnutrition on Host Defense and Infection. Clin Microbiol Rev. 2017;30:919–71. 28768707 10.1128/CMR.00119-16 PMC5608884

[48] van Vught AJAH, Dagnelie PC, Arts ICW, Froberg K, Andersen LB, El-Naaman B, Bugge A, Nielsen BM, Heitman BL. Dietary arginine and linear growth: the Copenhagen School Child Intervention Study. Br J Nutr. 2012;109:1031–9. 23046689 10.1017/S0007114512002942

[49] Semba RD, Shardell M, Sakr Ashour FA, Moaddel R, Trehan I, Maleta KM, Ordiz MI, Kraemer K, Khadeer MA, Ferrucci L, Manary MJ. Child Stunting is Associated with Low Circulating Essential Amino Acids. EBioMedicine. 2016;6:246–52. 27211567 10.1016/j.ebiom.2016.02.030 PMC4856740

[50] Karpiński M, Popko J, Maresz K, Badmaev V, Stohs SJ. Roles of Vitamins D and K, Nutrition, and Lifestyle in Low-Energy Bone Fractures in Children and Young Adults. J Am Coll Nutr. 2017;36:399–412. 28686548 10.1080/07315724.2017.1307791

[51] Kozioł-Kozakowska A, Maresz K. The Impact of Vitamin K2 (Menaquionones) in Children’s Health and Diseases: A Review of the Literature. Children (Basel). 2022;9:78. 35053702 10.3390/children9010078 PMC8774117

[52] Tsuchita H, Suzuki T, Kuwata T. The effect of casein phosphopeptides on calcium absorption from calcium-fortified milk in growing rats. Br J Nutr. 2001;85:5–10. 11227028 10.1079/bjn2000206

[53] Cao Y, Miao J, Liu G, Luo Z, Xia Z, Liu F, Yao M, Cao X, Sun S, Lin Y, et al. Bioactive Peptides Isolated from Casein Phosphopeptides Enhance Calcium and Magnesium Uptake in Caco-2 Cell Monolayers. J Agric Food Chem. 2017;65:2307–14. 28218527 10.1021/acs.jafc.6b05711

[54] Feng Y, Zhang J, Miao Y, Guo W, Feng G, Yang Y, Guo T, Wu H, Zeng M. Prevention of Zinc Precipitation with Calcium Phosphate by Casein Hydrolysate Improves Zinc Absorption in Mouse Small Intestine ex Vivo via a Nanoparticle-Mediated Mechanism. J Agric Food Chem. 2020;68:652–9. 31869222 10.1021/acs.jafc.9b07097

[55] Allen LH, Peerson JM, Olney DK. Provision of multiple rather than two or fewer micronutrients more effectively improves growth and other outcomes in micronutrient-deficient children and adults. J Nutr. 2009;139:1022–30. 19321586 10.3945/jn.107.086199

[56] Winzenberg T, Powell S, Shaw KA, Jones G. Effects of vitamin D supplementation on bone density in healthy children: systematic review and meta-analysis. BMJ. 2011;342:c7254. 21266418 10.1136/bmj.c7254 PMC3026600

[57] van Summeren MJH, van Coeverden SCCM, Schurgers LJ. Braam LAJLM, Noirt F, Uiterwaal CSPM, Kuis W, Vermeer C: Vitamin K status is associated with childhood bone mineral content. Br J Nutr. 2008;100:852–8. 18279558 10.1017/S0007114508921760

[58] Casey C, Kemp BJ, Cassidy L, Patterson CC, Tully MA, Hill AJ, McCance DR. The influence of diet and physical activity on bone density of children aged 5–7 years: The Belfast HAPO family study. Bone. 2023;172:116783. 37121559 10.1016/j.bone.2023.116783

[59] Soininen S, Sidoroff V, Lindi V, Mahonen A, Kröger L, Kröger H, Jääskeläinen J, Atalay M, Laaksonen DE, Laitinen T, Lakka TA. Body fat mass, lean body mass and associated biomarkers as determinants of bone mineral density in children 6–8years of age - The Physical Activity and Nutrition in Children (PANIC) study. Bone. 2018;108:106–14. 29307776 10.1016/j.bone.2018.01.003

[60] Chang Z, Lei W. A Study on the Relationship Between Physical Activity, Sedentary Behavior, and Sleep Duration in Preschool Children. Front Public Health. 2021;9:618962. 33898373 10.3389/fpubh.2021.618962 PMC8059703

[61] Ito T, Sugiura H, Ito Y, Noritake K, Ochi N. Relationship between the skeletal muscle mass index and physical activity of Japanese children: A cross-sectional, observational study. PLoS ONE. 2021;16:e0251025. 34038448 10.1371/journal.pone.0251025 PMC8153420

[62] Chaput JP, Gray CE, Poitras VJ, Carson V, Gruber R, Birken CS, MacLean JE, Aubert S, Sampson M, Tremblay MS. Systematic review of the relationships between sleep duration and health indicators in the early years (0–4 years). BMC Public Health. 2017;17:855. 29219078 10.1186/s12889-017-4850-2 PMC5773910

[63] Simoni P, Guglielmi R, Aparisi Gómez MP. Imaging of body composition in children. Quant Imaging Med Surg. 2020;10:1661–71. 32742959 10.21037/qims.2020.04.06 PMC7378095

[64] Crabtree NJ, Arabi A, Bachrach LK, Fewtrell M, El-Hajj Fuleihan G, Kecskemethy HH, Jaworski M, Gordon CM. Dual-energy X-ray absorptiometry interpretation and reporting in children and adolescents: the revised 2013 ISCD Pediatric Official Positions. J Clin Densitom. 2014;17:225–42. 24690232 10.1016/j.jocd.2014.01.003

[65] Pezzuti IL, Kakehasi AM, Filgueiras MT, de Guimarães JA, de Lacerda IAC, Silva IN. Imaging methods for bone mass evaluation during childhood and adolescence: an update. J Pediatr Endocrinol Metab. 2017;30:485–97. 28328530 10.1515/jpem-2016-0252

[66] Zhao Y, He L, Peng T, Liu L, Zhou H, Xu Y, Yang X, Huang Y, Chen Z, Xu Y. Nutritional status and function after high-calorie formula vs. Chinese food intervention in undernourished children with cerebral palsy. Front Nutr. 2022;9:960763. 36276835 10.3389/fnut.2022.960763 PMC9582948

