# Supplementary material for: Efficacy of long-term oral nutritional supplementation with dietary counseling on growth, body composition and bone mineralization in children with or at risk for undernutrition: a randomized controlled trial
Source: Nutr J. 2025 Jul 14;24:110. doi: 10.1186/s12937-025-01133-5 (PMC12257819; doi:10.1186/s12937-025-01133-5)
Supplement: Supplementary file 2 — Supplementary Material 1: Supplementary Tables S1-5, Supplementary Figures S1-2. Supplementary File S1. [file 12937_2025_1133_MOESM1_ESM.docx]

# SUPPLEMENTARY MATERIAL

**Table S1.** Nutrient composition in two servings of ONS and as a percentage of daily RNI for male Vietnamese children aged 3–5 years.

| **Nutrient** | **Amount in two servings (450 mL)** | **RNI for Vietnamese males aged 3–5 years,* %** |
| --- | --- | --- |
| Energy (kcal) | 452 | 34.2 |
| Protein (g) | 13.48 | 53.9 |
| Lipid (g) | 17.62 | 40.5 |
| Carbohydrate (g) | 58.94 | 30.2 |
| Calcium (mg) | 450 | 75.0 |
| Iron (mg) | 6.3 | 114.5 |
| Zinc (mg) | 3.02 | 62.9 |
| Vitamin A (μg RE) | 270 | 54.0 |
| Vitamin D (μg) | 9.0 | 60.0 |
| Vitamin E (IU) | 10.4 | 231.1 |
| Vitamin B1 (mg) | 1.1 | 157.1 |
| Vitamin B2 (mg) | 0.96 | 120.0 |
| Niacin (mg) | 6.74 | 84.3 |
| Pantothenic acid (mg) | 3.14 | 104.7 |
| Vitamin B6 (mg) | 1.18 | 236.0 |
| Folic acid (μg) | 108 | 122.4 |
| Vitamin B12 (μg) | 1.36 | 136.0 |
| Vitamin C (mg) | 45.0 | 112.5 |
| Vitamin K (total) (μg) | 34.6 | N/A |
| Vitamin K1 (phylloquinone) (μg) | 26.6 | N/A |
| Vitamin K2 (menaquinone) (μg) | 8.0 | N/A |

*Age- and sex-specific 2016 Vietnamese RNI.^62^

N/A, not applicable; ONS, oral nutritional supplement; RE, retinol equivalent; RNI, recommended nutrient intake

**Table S2.** Supportive exploratory outcomes and adverse events collection in the SPROUT study.

| **Outcome** | **Description** |
| --- | --- |
| HAD and WAD^1,2^ | - HAD: Difference between measured height and the median sex-age-specific height using WHO Growth Standards - WAD: Difference between measured weight and the median sex-age-specific weight using WHO Growth Standards |
| Nutritional status^2,3^ | Defined as z-score indices <−3 (severe), ≥−3 to <−2 (moderate), ≥−2 to <−1 (mild), and ≥−1 (normal) for underweight (WAZ), stunting (HAZ), and wasting (WHZ), according to the WHO Growth Standards. |
| Arm anthropometry^4,5^ | Measurements   - Mid-upper arm circumference (MUAC) measured with a flexible, nonstretchable measuring tape - Triceps skinfold (TSF) measured with skinfold caliper (Harpenden)   Calculation of indices   - Mid-upper-arm muscle circumference (MUAMC) = MUAC – (TSF × π); - Arm muscle area (AMA) = $\frac{\left( \mathbf{MUAC}\boldsymbol{-}\mathbf{TSF}\boldsymbol{\times\pi} \right)^{\boldsymbol{2}}}{\boldsymbol{4}\boldsymbol{\pi}}$ - Arm fat area (AFA) = $\frac{\boldsymbol{MUAC}^{\boldsymbol{2}}}{\boldsymbol{4\pi}}- AMA$ - Arm fat index (AFI) = AFA / $\frac{\boldsymbol{MUAC}^{\boldsymbol{2}}}{\boldsymbol{4}\boldsymbol{\pi}}$ |
| Lower leg length | Measured with knemometer (Shorr Knee-Height Caliper) |
| Total body lean mass,  fat mass, and their  height-normalized indices | - Assessed by DXA using the OsteoSys PRIMUS densitometer (OsteoSys, Seoul, Korea) - Lean and fat mass index: calculated as mass (kg)/height (m^2^) |
| BMC and BMD of TBLH, AP lumbar spine (L1–L4), and bilateral total hip^6^ | Assessed by DXA using the OsteoSys PRIMUS densitometer (OsteoSys, Seoul, Korea) |
| BMAD of the AP lumbar spine (L1–L4)^7^ | Assessed by DXA using the OsteoSys PRIMUS densitometer (OsteoSys, Seoul, Korea)   - BMAD was calculated per Kindler et al. (2019) |
| Handgrip strength for children aged ≥36 months | Assessed using the digital Jamar handgrip strength dynamometer |
| Fasting nutritional and bone health blood biomarkers | - Capillary blood hemoglobin (HemoCue Hb 301) - Total serum 25-hydroxyvitamin D (LC-MS/MS) - Serum albumin (Abbott Architect c4000 chemistry analyzer) - Serum uCoC (Takara Bio Inc.) - Serum CoC (Eagle Biosciences) - uCOC:CoC ratio as an indicator of vitamin K status - Serum amino acids (UPLC waters) |
| Illness episodes, sick days, missed school days, missed parent workdays due to child’s illness, and  healthcare expenditure | Assessed with a parent-reported illness questionnaire  Covariates known to affect child immunity evaluated for inclusion in multivariable models: duration of exclusive breastfeeding, duration of all (exclusive and partial) breastfeeding, number of children 13 years old and under in the household, any smoking in the household, number of smokers in the household, mother’s highest education, father’s highest education, baseline height-for-age, weight-for-age and weight-for-height z-scores, annual household annual income. |
| Attentional focus^8,9^ | Assessed using the attentional focusing subscale of the ECBQ for children aged 24–36 months at baseline and the CBQ for children aged >36 months at baseline |
| Sleep patterns | Assessed with parent-reported sleep questionnaire:   - Daytime sleep duration - Nighttime sleep duration - Sleep quality on a 10-point VAS - Number of night sleep awakenings - Duration of night sleep awakenings |
| Physical activity, energy, and appetite levels | Assessed with parent-reported 10-point VAS |
| Parental satisfaction with the child’s health | Assessed with parent-reported 10-point VAS |
| Adverse events | Collected by a standard method where all untoward medical occurrences temporally associated with the study, whether or not related to the study product, were recorded. AEs were reported by parents and caregivers and recorded by the study sites. Both nonserious and serious AEs were medically confirmed and assessed for causality by the study physicians. AEs were coded and grouped according to the Medical Dictionary for Regulatory Activities (MedRA version 21.1) terminology. |

AE, adverse event; AP, anteroposterior; BMAD, bone mineral apparent density; BMC, bone mineral content; BMD, bone mineral density; CBQ, Children’s Behavioral Questionnaire; CoC, carboxylated osteocalcin; DXA, dual-energy X-ray absorptiometry; ECBQ, Early Childhood Behavior Questionnaire; HAD, height-for-age difference; HAZ, height-for-age z-score; LC-MS/MS, liquid chromatography tandem mass spectrometry; SPROUT, Supporting Pediatric Growth and Health Outcomes; TBLH, total body less head; uCoC, undercarboxylated osteocalcin; VAS, visual analog scale; WAD, weight-for-age difference; WAZ, weight-for-age z-score; WHO, World Health Organization; WHZ, weight-for-height z-score

**References:** **1.** Leroy JL, Ruel M, Habicht JP, Frongillo EA. Using height-for-age differences (HAD) instead of height-for-age z-scores (HAZ) for the meaningful measurement of population-level catch-up in linear growth in children less than 5 years of age. *BMC Pediatr*. 2015;15:145. doi:10.1186/s12887-015-0458-9. **2.** World Health Organization. WHO child growth standards: length/height-for-age, weight-for-age, weight-for-length, weight-for-height and body mass index-for-age: methods and development: World Health Organization; 2006. **3.** Mehta NM, Corkins MR, Lyman B, et al. Defining pediatric malnutrition: a paradigm shift toward etiology‐related definitions. *JPEN J Parenter Enteral Nutr*. 2013;37:460-481. doi:10.1177/0148607113479972. **4.** Gibson RS. Principles of nutritional assessment: Oxford university press, USA; 2005. **5.** Frisancho AR. Anthropometric standards for the assessment of growth and nutritional status: University of Michigan Press; 1990. **6.** Crabtree NJ, Arabi A, Bachrach LK, et al. Dual-energy X-ray absorptiometry interpretation and reporting in children and adolescents: the revised 2013 ISCD Pediatric Official Positions. *J Clin Densitom*. 2014;17:225-242. doi:10.1016/j.jocd.2014.01.003. **7.** Kindler JM, Lappe JM, Gilsanz V, et al. Lumbar spine bone mineral apparent density in children: results from the bone mineral density in childhood study. *J Clin Endocrinol Metab*. 2019;104:1283-1292. doi:10.1210/jc.2018-01693. **8.** Putnam SP, Gartstein MA, Rothbart MK. Measurement of fine-grained aspects of toddler temperament: the Early Childhood Behavior Questionnaire. *Infant Behav Dev*. 2006;29:386-401. doi:10.1016/j.infbeh.2006.01.004. **9.** Rothbart MK, Ahadi SA, Hershey KL, Fisher P. Investigations of temperament at three to seven years: the Children's Behavior Questionnaire. Child Dev. 2001;72:1394-408.

**Table S3.** Change in anthropometric parameters from baseline to days 30, 120, and 240.*

|  | **Visits** | **ONS + DC** | **DC only** | **Difference** | ***P*-value** |
| --- | --- | --- | --- | --- | --- |
| **Weight (kg)** | Baseline | 12.51 (0.07) | 12.59 (0.07) | –0.08 (0.09) | 0.37 |
|  | Change at Day 30 | 0.64 (0.03) | 0.44 (0.03) | 0.21 (0.05) | **< 0.001** |
|  | Change at Day 120 | 1.04 (0.04) | 0.73 (0.04) | 0.31 (0.05) | **< 0.001** |
|  | Change at Day 240 | 1.51 (0.05) | 1.05 (0.05) | 0.46 (0.07) | **< 0.001** |
| **WAZ** | Baseline | –1.91 (0.05) | –1.85 (0.04) | –0.06 (0.06) | 0.37 |
|  | Change at Day 30 | 0.30 (0.02) | 0.18 (0.02) | 0.12 (0.03) | **< 0.001** |
|  | Change at Day 120 | 0.30 (0.02) | 0.13 (0.02) | 0.17 (0.03) | **< 0.001** |
|  | Change at Day 240 | 0.25 (0.03) | 0.00 (0.03) | 0.24 (0.04) | **< 0.001** |
| **WAP** | Baseline | 4.39 (0.32) | 4.91 (0.32) | –0.53 (0.43) | 0.23 |
|  | Change at Day 30 | 3.38 (0.27) | 1.84 (0.26) | 1.54 (0.36) | **< 0.001** |
|  | Change at Day 120 | 3.55 (0.30) | 1.49 (0.29) | 2.06 (0.40) | **< 0.001** |
|  | Change at Day 240 | 3.68 (0.44) | 0.28 (0.42) | 3.41 (0.58) | **< 0.001** |
| **WAD (kg)** | Baseline | –3.43 (0.07) | –3.35 (0.07) | –0.08 (0.09) | 0.65 |
|  | Change at Day 30 | 0.43 (0.03) | 0.22 (0.03) | 0.20 (0.05) | **< 0.001** |
|  | Change at Day 120 | 0.32 (0.04) | 0.01 (.0.04) | 0.31 (0.05) | **< 0.001** |
|  | Change at Day 240 | 0.08 (0.05) | –0.38 (0.05) | 0.46 (0.07) | **< 0.001** |
| **Height (cm)** | Baseline | 94.28 (0.18) | 94.17 (0.18) | 0.11 (0.25) | 0.66 |
|  | Change at Day 30 | 0.87 (0.06) | 0.85 (0.06) | 0.02 (0.08) | 0.83 |
|  | Change at Day 120 | 2.98 (0.07) | 2.52 (0.07) | 0.46 (0.09) | **< 0.001** |
|  | Change at Day 240 | 5.42 (0.07) | 4.68 (0.07) | 0.73 (0.10) | **< 0.001** |
| **HAZ** | Baseline | –1.84 (0.04) | –1.86 (0.04) | 0.02 (0.06) | 0.73 |
|  | Change at Day 30 | 0.05 (0.01) | 0.05 (0.01) | 0.00 (0.02) | 0.85 |
|  | Change at Day 120 | 0.20 (0.02) | 0.09 (0.02) | 0.11 (0.02) | **< 0.001** |
|  | Change at Day 240 | 0.29 (0.02) | 0.13 (0.02) | 0.17 (0.02) | **< 0.001** |
| **HAP** | Baseline | 4.89 (0.34) | 4.79 (0.33) | 0.10 (0.45) | 0.82 |
|  | Change at Day 30 | 0.75 (0.22) | 0.72 (0.21) | 0.03 (0.29) | 0.93 |
|  | Change at Day 120 | 2.66 (0.26) | 1.11 (0.26) | 1.54 (0.35) | **< 0.001** |
|  | Change at Day 240 | 4.09 (0.30) | 1.44 (0.29) | 2.66 (0.40) | **< 0.001** |
| **HAD (cm)** | Baseline | –7.65 (0.18) | –7.76 (0.18) | 0.11 (0.24) | 0.41 |
|  | Change at Day 30 | 0.12 (0.06) | 0.11 (0.06) | 0.01 (0.08) | 0.84 |
|  | Change at Day 120 | 0.56 (0.07) | 0.10 (0.07) | 0.46 (0.09) | **< 0.001** |
|  | Change at Day 240 | 0.70 (0.08) | –0.03 (.0.07) | 0.73 (0.10) | **< 0.001** |
| **WHZ** | Baseline | –1.23 (0.05) | –1.12 (0.05) | –0.10 (0.07) | 0.13 |
|  | Change at Day 30 | 0.40 (0.03) | 0.24 (0.03) | 0.16 (0.04) | **0.001** |
|  | Change at Day 120 | 0.31 (0.04) | 0.15 (0.04) | 0.16 (0.05) | **0.002** |
|  | Change at Day 240 | 0.14 (0.05) | –0.05 (0.04) | 0.19 (0.06) | **0.002** |
| **WHP** | Baseline | 14.50 (0.93) | 16.50 (0.92) | –2.01 (1.25) | 0.11 |
|  | Change at Day 30 | 10.16 (0.81) | 5.82 (0.80) | 4.34 (1.09) | **< 0.001** |
|  | Change at Day 120 | 7.68 (0.88) | 4.08 (0.85) | 3.60 (1.16) | **0.002** |
|  | Change at Day 240 | 5.20 (1.20) | –0.16 (1.13) | 5.37 (1.57) | **< 0.001** |
| **BMIAZ** | Baseline | –1.07 (0.05) | –0.96 (0.05) | –0.11 (0.07) | 0.11 |
|  | Change at Day 120 | 0.38 (0.03) | 0.23 (0.03) | 0.16 (0.04) | **0.001** |
|  | Change at Day 120 | 0.24 (0.04) | 0.10 (0.04) | 0.14 (0.05) | **0.006** |
|  | Change at Day 240 | 0.07 (0.04) | –0.11 (0.04) | 0.18 (0.05) | **< 0.001** |
| **BMIAP** | Baseline | 17.87 (1.07) | 20.20 (1.05) | –2.33 (1.43) | 0.11 |
|  | Change at Day 30 | 10.70 (0.87) | 5.98 (0.85) | 4.72 (1.17) | **< 0.001** |
|  | Change at Day 120 | 6.59 (0.92) | 3.02 (0.90) | 3.58 (1.24) | **0.004** |
|  | Change at Day 240 | 3.00 (1.02) | –1.97 (0.99) | 4.97 (1.36) | **< 0.001** |
| **MUACZ** | Baseline | –1.23 (0.05) | –1.19 (0.05) | –0.04 (0.07) | 0.53 |
|  | Change at Day 30 | 0.05 (0.03) | –0.06 (0.03) | 0.11 (0.04) | **0.006** |
|  | Change at Day 120 | 0.06 (0.04) | –0.04 (0.03) | 0.10 (0.05) | **0.03** |
|  | Change at Day 240 | 0.32 (0.05) | 0.11 (0.04) | 0.21 (0.06) | **<0.001** |
| **MUACP** | Baseline | 14.20 (1.00) | 15.33 (0.98) | –1.13 (1.34) | 0.40 |
|  | Change at Day 30 | 0.89 (0.57) | –0.97 (0.57) | 1.86 (0.77) | **0.02** |
|  | Change at Day 120 | 1.30 (0.76) | –0.46 (0.73) | 1.76 (1.00) | 0.08 |
|  | Change at Day 240 | 7.92 (1.26) | 2.41 (1.20) | 5.51 (1.66) | **0.001** |

Data are presented as LSM (SE).
Baseline values: ANOVA. Change values: ANCOVA.
**P*-values in bold are *P* < 0.05, underlined *P*-values are *P* < 0.10 and > 0.05.

ANCOVA, analysis of covariance; ANOVA, analysis of variance; BMIAP, body mass index-for-age percentile; BMIAZ, body mass index-for-age z-score; DC, dietary counseling; HAD, height-for-age difference; HAP, height-for-age percentile; HAZ, height-for-age z-score; LSM, least squares mean; MUACP, mid-upper arm circumference percentile; MUACZ, mid-upper arm circumference z-score; ONS, oral nutritional supplement; SE, standard error; WAD, weight-for-age difference; WAP, weight-for-age percentile; WAZ, weight-for-age z-score; WHP, weight-for-height percentile; WHZ, weight-for-height z-score

**Table S4.** Individual participant data listing of participants with a low hemoglobin level (< 11 g/dL) at any time point (baseline or day 240)

| **Subject ID** | **Treatment group** | **Visit** | **Hemoglobin (g/dL)** | **Hemoglobin Status** | **Status change from baseline to day 230** |
| --- | --- | --- | --- | --- | --- |
| 3339028 | DC only | Baseline | 10.7 | Low; Mild -10.0 - <= 11 g/dL | Recovered |
| 3339028 | DC only | Day 240 | 11.6 | Normal (> 11 g/dL) |  |
| 3340003 | DC only | Baseline | 10.6 | Low; Mild -10.0 - <= 11 g/dL | Recovered |
| 3340003 | DC only | Day 240 | 11.5 | Normal (> 11 g/dL) |  |
| 3338017 | ONS+DC | Baseline | 10.3 | Low; Mild -10.0 - <= 11 g/dL | Recovered |
| 3338017 | ONS+DC | Day 240 | 13.4 | Normal (> 11 g/dL) |  |
| 3342025 | ONS+DC | Baseline | 10.4 | Low; Mild -10.0 - <= 11 g/dL | Recovered |
| 3342025 | ONS+DC | Day 240 | 11.0 | Normal (> 11 g/dL) |  |
| 3340050 | ONS+DC | Baseline | 10.7 | Low; Mild -10.0 - <= 11 g/dL | No change |
| 3340050 | ONS+DC | Day 240 | 10.7 | Low; Mild -10.0 - <= 11 g/dL |  |
| 3339073 | DC only | Baseline | 11.4 | Normal (> 11 g/dL) | Incident |
| 3339073 | DC only | Day 240 | 10.8 | Low; Mild -10.0 - <= 11 g/dL |  |
| 3342027 | DC only | Baseline | 11.4 | Normal (> 11 g/dL) | Incident |
| 3342027 | DC only | Day 240 | 10.9 | Low; Mild -10.0 - <= 11 g/dL |  |
| 3337006 | ONS+DC | Baseline | 11.4 | Normal (> 11 g/dL) | Incident |
| 3337006 | ONS+DC | Day 240 | 10.8 | Low; Mild -10.0 - <= 11 g/dL |  |
| 3337018 | ONS+DC | Baseline | 11.3 | Normal (> 11 g/dL) | Incident |
| 3337018 | ONS+DC | Day 240 | 10.9 | Low; Mild -10.0 - <= 11 g/dL |  |
| 3339070 | ONS+DC | Baseline | 11.5 | Normal (> 11 g/dL) | Incident |
| 3339070 | ONS+DC | Day 240 | 10.6 | Low; Mild -10.0 - <= 11 g/dL |  |
| 3339071 | ONS+DC | Baseline | 11.6 | Normal (> 11 g/dL) | Incident |
| 3339071 | ONS+DC | Day 240 | 10.1 | Low; Mild -10.0 - <= 11 g/dL |  |
| 3342017 | ONS+DC | Baseline | 11.7 | Normal (> 11 g/dL) | Incident |
| 3342017 | ONS+DC | Day 240 | 10.4 | Low; Mild -10.0 - <= 11 g/dL |  |

**Table S5.** Parent-reported child appetite, physical activity, energy levels, and sleep habits – overall treatment effect across post-baseline visits

| **Sleep** | **Visit** | **ONS + DC** | **DC only** | **Difference** | ***P*-value** |
| --- | --- | --- | --- | --- | --- |
| Appetite  (10-point VAS) | Baseline | 6.14 (0.13) | 5.95 (0.13) | 0.19 (0.17) | 0.27 |
|  | Day 30/120/240 | 6.99 (0.07) | 6.41 (0.07) | 0.58 (0.10) | **< 0.001** |
| Physical activity  (10-point VAS) | Baseline | 7.79 (0.11) | 7.85 (0.11) | –0.07 (0.14) | 0.65 |
|  | Day 30/120/240 | 8.15 (0.06) | 7.83 (0.05) | 0.31 (0.07) | **< 0.001** |
| Energy  (10-point VAS) | Baseline | 7.76 (0.12) | 7.82 (0.12) | –0.06 (0.16) | 0.70 |
|  | Day 30/120/240 | 8.20 (0.10) | 7.90 (0.10) | 0.27 (0.08) | **< 0.001** |
| Number night awakenings per night^a^ | Baseline | 0.5 (0.1) | 0.5 (0.1) | NS | 0.05 |
|  | Day 30 | 0.3 (0.1) | 0.4 (0.1) |  |  |
|  | Day 120 | 0.2 (0.1) | 0.3 (0.1) |  |  |
|  | Day 240 | 0.3 (0.1) | 0.2 (0.0) |  |  |
| Duration of night awakening (mins) | Baseline | 2.29 (0.48) | 2.10 (0.47) | 0.20 (0.65) | – |
|  | Day 30/120/240 | 0.94 (0.20) | 1.77 (0.20) | –0.83 (0.27) | **0.002** |
| Total night sleep (hours) | Baseline | 9.18 (0.06) | 9.22 (0.06) | –0.04 (0.08) | – |
|  | Day 30/120/240 | 9.21 (0.04) | 9.09 (0.04) | 0.12 (0.06) | **0.03** |
| Overall sleep quality  (10-point VAS) | Baseline | 7.97 (0.11) | 8.17 (0.10) | –0.20 (0.14) | – |
|  | Day 30/120/240 | 8.69 (0.05) | 8.38 (0.05) | 0.31 (0.07) | **< 0.001** |
| Total day sleep (hours) | Baseline | 1.87 (0.04) | 1.83 (0.04) | 0.04 (0.05) | – |
|  | Day 30/120/240 | 1.85 (0.03) | 1.89 (0.04) | –0.04 (0.05) | 0.44 |

LSM (SE) of the overall treatment effect across day 30, 120 and 240, unless otherwise stated. Day 30/120/240 analyses: Repeated-measures ANCOVA (site, treatment, gender, treatment*gender, visit, treatment*visit and with age [in months] and respective baseline variable as covariates).

*P*-values in bold are *P* < 0.05.

^a^Observed mean (SE) for each visit. Baseline analysis: chi-square test; and day 30/120/240 analysis: GEE (site, treatment, gender, treatment*gender, visit, treatment*visit, with age [in months] and baseline value as covariates).

ANCOVA, analysis of covariance; DC, dietary counseling; GEE, generalized estimating equation; LSM, least squares mean; NS, Not significant; ONS, oral nutritional supplement; SE, standard error; VAS, visual analog scale

**Figure S1.** Parent-rated child health outcomes at baseline and days 30, 120, and 240 by treatment group for **(A)** physical activity^a^, **(B)** energy/activity^a,b^ levels, and **(C)** appetite^a^.

^a^Indicates *P* < 0.05 for the overall treatment effect between groups with ANCOVA across post-baseline timepoints.

^b^Energy/Activity: Parents were asked to “indicate your child’s energy/activity level, as indicated by being alert, curious, and eager to explore and learn.” Baseline values are ANOVA LSM (SE), and day 30, 120, and 240 values are from repeated-measures ANCOVA estimates.

*Indicates *P* < 0.05 for between-group comparisons with ANCOVA for that timepoint.

ANCOVA, analysis of covariance; ANOVA, analysis of variance; DC, dietary counseling; LSM, least squares mean; ONS, oral nutritional supplement; SE, standard error; VAS, visual analog scale

**(A)**


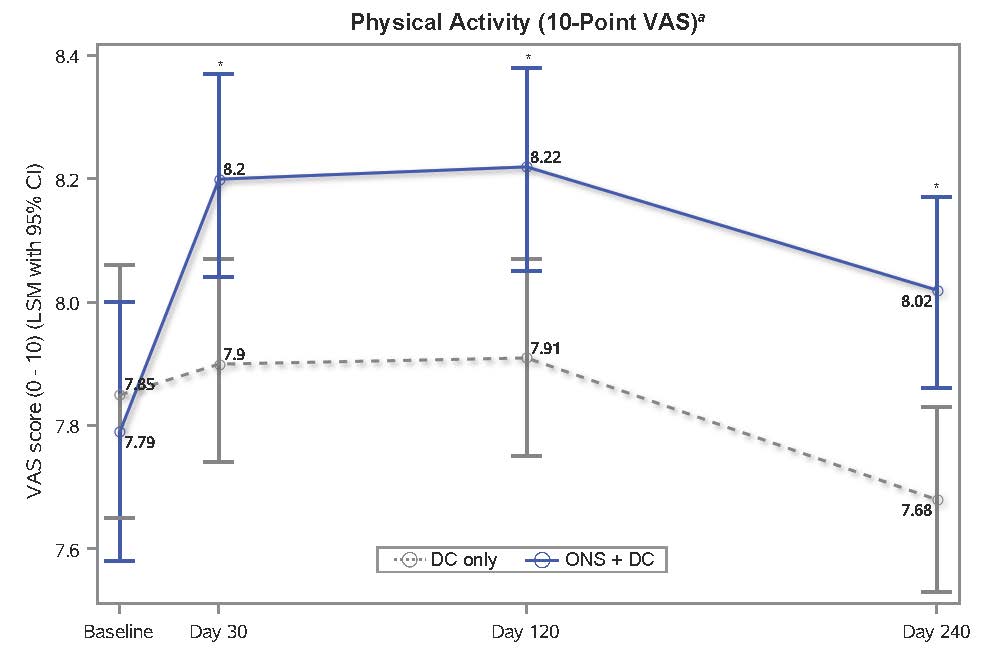


**(B)**


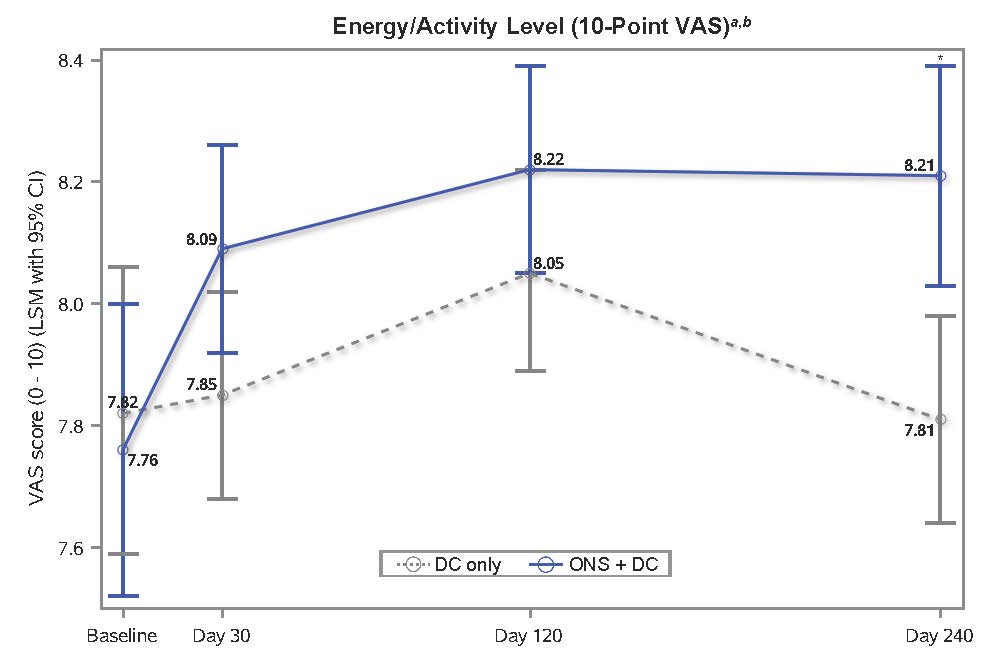


**(C)**


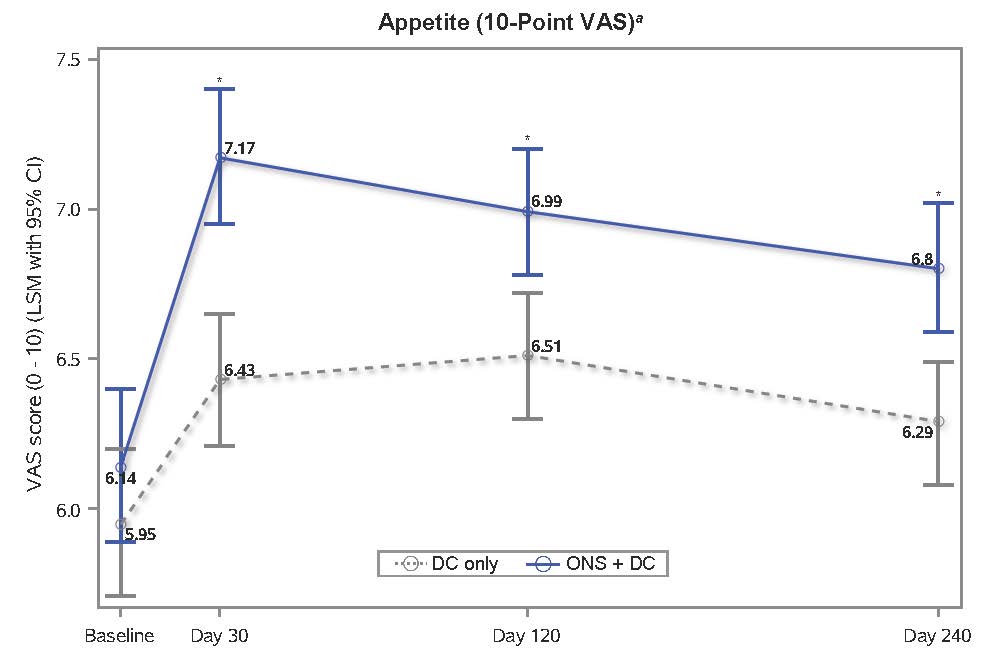


**Figure S2.** Parent-rated child sleep habits at baseline and day 30, 120, and 240 by treatment group for **(A)** duration of night awakenings, **(B)** night sleep duration, **(C)** sleep quality rated on a 10-point VAS, and **(D)** day sleep duration.

Baseline values are ANOVA LSM (SE), and day 30, 120, and 240 values are from repeated-measures ANCOVA estimates.

^a^Indicates *P* < 0.05 for the overall treatment effect between groups with ANCOVA across post-baseline timepoints.

^b^Indicates *P* < 0.05 for the treatment-by-visit interaction effect over the post-baseline visits.

*Indicates *P* < 0.05 for between-group comparisons with ANCOVA for that timepoint.

ANCOVA, analysis of covariance; ANOVA, analysis of variance; DC, dietary counseling; LSM, least squares mean; ONS, oral nutritional supplement; SE, standard error; VAS, visual analog scale

**(A)**


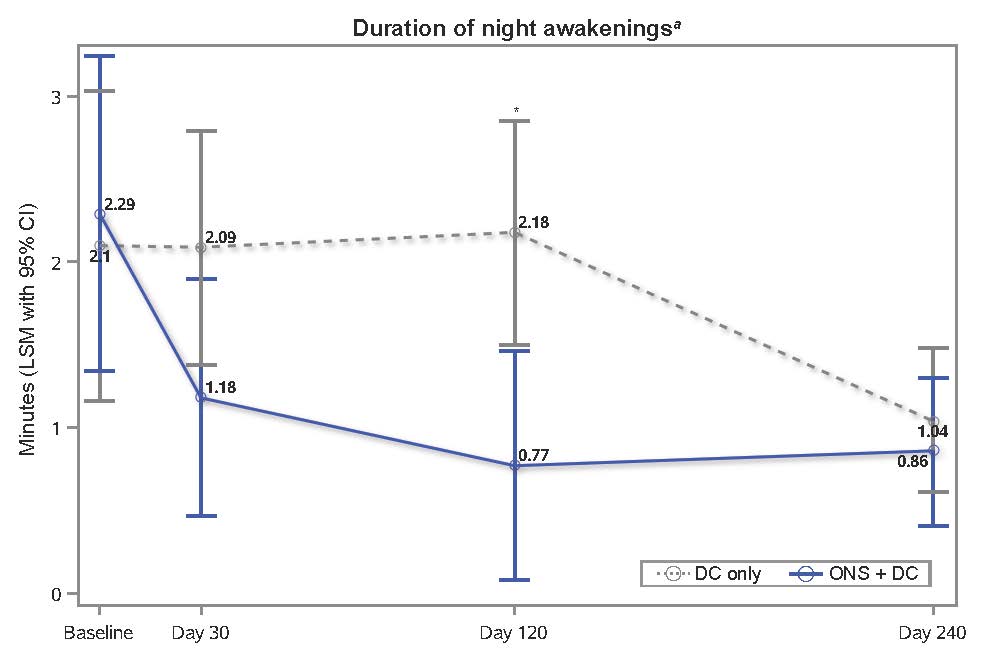


**(B)**


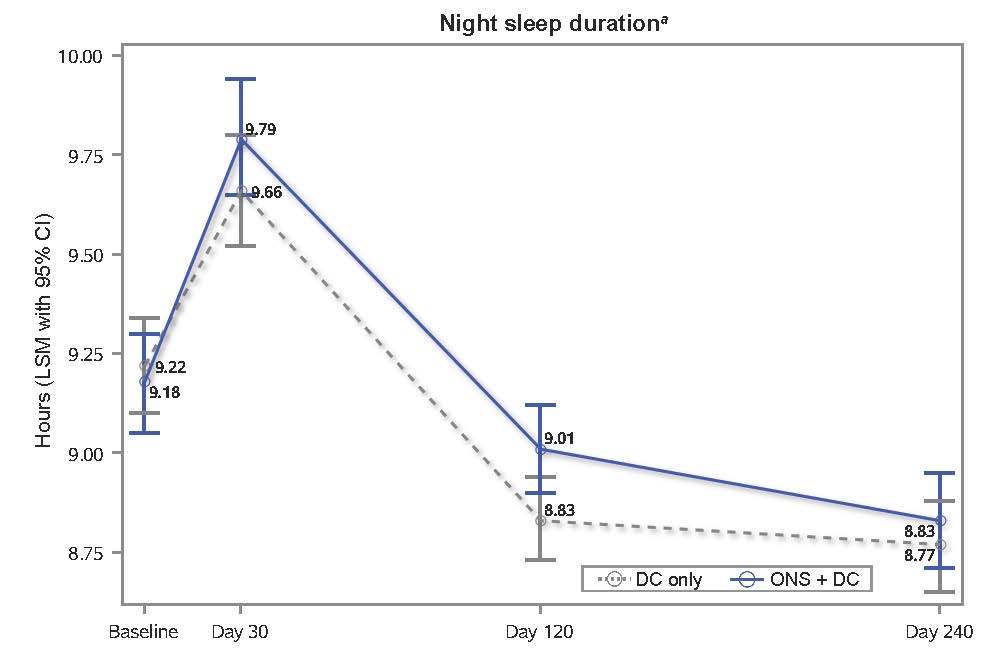


**(C)**


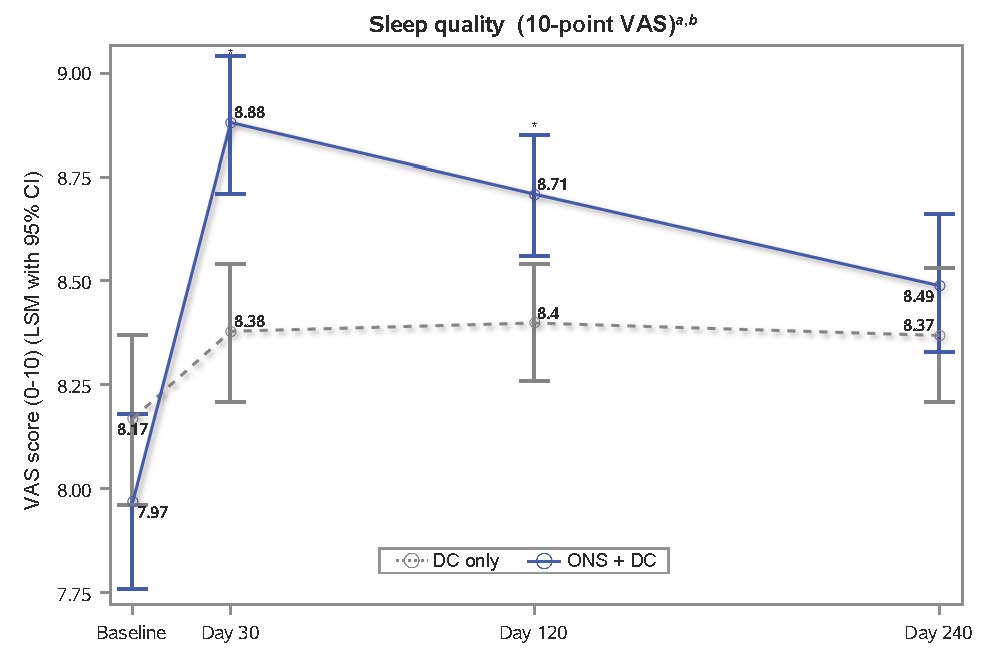


**(D)**


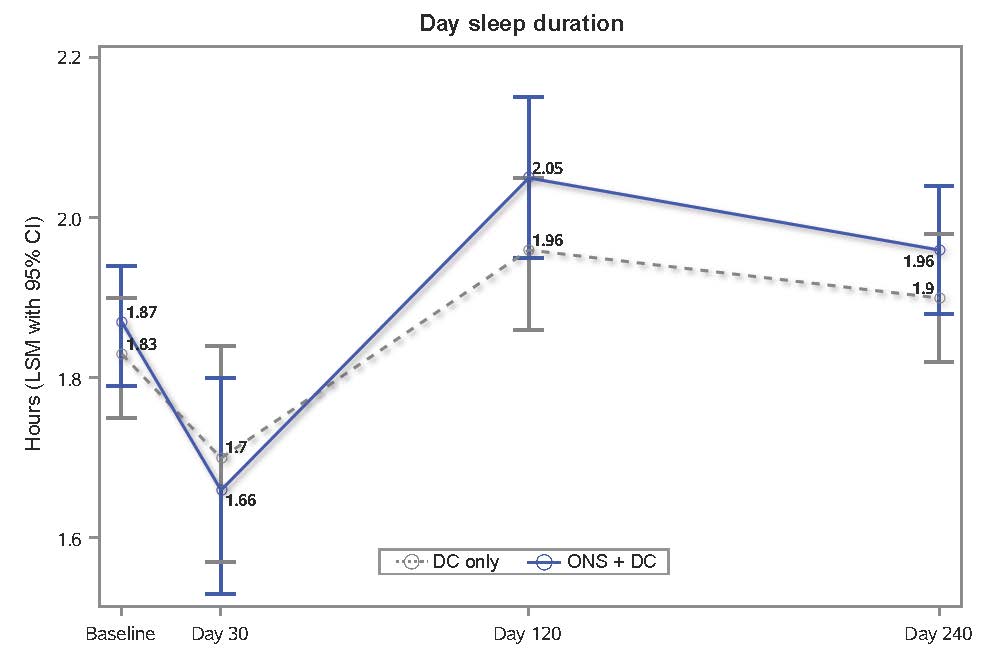


Supplementary File S1. CONSORT 2010 checklist of information to include when reporting a randomised trial*

| Section/Topic | Item No | Checklist item | Reported on page No |
| --- | --- | --- | --- |
| Title and abstract | | | |
|  | 1a | Identification as a randomised trial in the title | 1 |
|  | 1b | Structured summary of trial design, methods, results, and conclusions (for specific guidance see CONSORT for abstracts) | 2 |
| Introduction | | | |
| Background and objectives | 2a | Scientific background and explanation of rationale | 3-5 |
|  | 2b | Specific objectives or hypotheses | 5 |
| Methods | | | |
| Trial design | 3a | Description of trial design (such as parallel, factorial) including allocation ratio | 6-7 |
|  | 3b | Important changes to methods after trial commencement (such as eligibility criteria), with reasons | N/A |
| Participants | 4a | Eligibility criteria for participants | 6 |
|  | 4b | Settings and locations where the data were collected | 6 |
| Interventions | 5 | The interventions for each group with sufficient details to allow replication, including how and when they were actually administered | 7 |
| Outcomes | 6a | Completely defined pre-specified primary and secondary outcome measures, including how and when they were assessed | 7 and Table S2 |
|  | 6b | Any changes to trial outcomes after the trial commenced, with reasons | N/A |
| Sample size | 7a | How sample size was determined | 8 |
|  | 7b | When applicable, explanation of any interim analyses and stopping guidelines | N/A |
| Randomisation: |  |  |  |
| Sequence generation | 8a | Method used to generate the random allocation sequence | 6 |
|  | 8b | Type of randomisation; details of any restriction (such as blocking and block size) | 6 |
| Allocation concealment mechanism | 9 | Mechanism used to implement the random allocation sequence (such as sequentially numbered containers), describing any steps taken to conceal the sequence until interventions were assigned | 6 |
| Implementation | 10 | Who generated the random allocation sequence, who enrolled participants, and who assigned participants to interventions | 6 |
| Blinding | 11a | If done, who was blinded after assignment to interventions (for example, participants, care providers, those assessing outcomes) and how | N/A: open-label |
|  | 11b | If relevant, description of the similarity of interventions | N/A: open-label |
| Statistical methods | 12a | Statistical methods used to compare groups for primary and secondary outcomes | 8-9 |
|  | 12b | Methods for additional analyses, such as subgroup analyses and adjusted analyses | 9 |
| Results | | | |
| Participant flow (a diagram is strongly recommended) | 13a | For each group, the numbers of participants who were randomly assigned, received intended treatment, and were analysed for the primary outcome | 10 & Fig 1 |
|  | 13b | For each group, losses and exclusions after randomisation, together with reasons | Fig 1 |
| Recruitment | 14a | Dates defining the periods of recruitment and follow-up | 6 |
|  | 14b | Why the trial ended or was stopped | N/A |
| Baseline data | 15 | A table showing baseline demographic and clinical characteristics for each group | Pg 11, Table 1 |
| Numbers analysed | 16 | For each group, number of participants (denominator) included in each analysis and whether the analysis was by original assigned groups | 10 |
| Outcomes and estimation | 17a | For each primary and secondary outcome, results for each group, and the estimated effect size and its precision (such as 95% confidence interval) | 12-22, Tables 2-7 |
|  | 17b | For binary outcomes, presentation of both absolute and relative effect sizes is recommended | Table 4, 7 |
| Ancillary analyses | 18 | Results of any other analyses performed, including subgroup analyses and adjusted analyses, distinguishing pre-specified from exploratory | 9 |
| Harms | 19 | All important harms or unintended effects in each group (for specific guidance see CONSORT for harms) | 23 |
| Discussion | | | |
| Limitations | 20 | Trial limitations, addressing sources of potential bias, imprecision, and, if relevant, multiplicity of analyses | 27 |
| Generalisability | 21 | Generalisability (external validity, applicability) of the trial findings | 24-25 |
| Interpretation | 22 | Interpretation consistent with results, balancing benefits and harms, and considering other relevant evidence | 24-27 |
| Other information | | |  |
| Registration | 23 | Registration number and name of trial registry | 3 |
| Protocol | 24 | Where the full trial protocol can be accessed, if available | N/A |
| Funding | 25 | Sources of funding and other support (such as supply of drugs), role of funders | 29 |

Citation: Schulz KF, Altman DG, Moher D, for the CONSORT Group. CONSORT 2010 Statement: updated guidelines for reporting parallel group randomised trials. BMC Medicine. 2010;8:18.
© 2010 Schulz et al. This is an Open Access article distributed under the terms of the Creative Commons Attribution License (<http://creativecommons.org/licenses/by/2.0>), which permits unrestricted use, distribution, and reproduction in any medium, provided the original work is properly cited.

*We strongly recommend reading this statement in conjunction with the CONSORT 2010 Explanation and Elaboration for important clarifications on all the items. If relevant, we also recommend reading CONSORT extensions for cluster randomised trials, non-inferiority and equivalence trials, non-pharmacological treatments, herbal interventions, and pragmatic trials. Additional extensions are forthcoming: for those and for up-to-date references relevant to this checklist, see [www.consort-statement.org](http://www.consort-statement.org).
